# Approximate planning in spatial search

**DOI:** 10.1371/journal.pcbi.1012582

**Published:** 2024-11-12

**Authors:** Marta Kryven, Suhyoun Yu, Max Kleiman-Weiner, Tomer Ullman, Joshua Tenenbaum

**Affiliations:** 1 Department of Brain and Cognitive Sciences, Massachusetts Institute of Technology, Cambridge, Massachusetts, United States of America; 2 Department of Mechanical Engineering, Massachusetts Institute of Technology, Cambridge, Massachusetts, United States of America; 3 Department of Psychology, Harvard University, Cambridge, Massachusetts, United States of America; Max Planck Institute for Evolutionary Biology: Max-Planck-Institut fur Evolutionsbiologie, GERMANY

## Abstract

How people plan is an active area of research in cognitive science, neuroscience, and artificial intelligence. However, tasks traditionally used to study planning in the laboratory tend to be constrained to artificial environments, such as Chess and bandit problems. To date there is still no agreed-on model of how people plan in realistic contexts, such as navigation and search, where values intuitively derive from interactions between perception and cognition. To address this gap and move towards a more naturalistic study of planning, we present a novel spatial Maze Search Task (MST) where the costs and rewards are physically situated as distances and locations. We used this task in two behavioral experiments to evaluate and contrast multiple distinct computational models of planning, including optimal expected utility planning, several one-step heuristics inspired by studies of information search, and a family of planners that deviate from optimal planning, in which action values are estimated by the interactions between perception and cognition. We found that people’s deviations from optimal expected utility are best explained by planners with a limited horizon, however our results do not exclude the possibility that in human planning action values may be also affected by cognitive mechanisms of numerosity and probability perception. This result makes a novel theoretical contribution in showing that limited planning horizon generalizes to spatial planning, and demonstrates the value of our multi-model approach for understanding cognition.

## Author summary

We present a computational study of spatial planning under uncertainty using a novel Maze Search Task (MST), in which people search mazes for probabilistically hidden rewards. The MST is designed to resemble real-life spatial planning, where costs and rewards are physically situated as distances and locations. We found that people’s spatial planning is best explained by planners with limited planning horizon, as opposed to both myopic heuristics or the optimal expected utility, showing that a limited planning horizon can generalize to spatial planning tasks. We also find that our results do not exclude the possibility that in human planning action values may be affected by cognitive mechanisms of numerosity and probability perception.

## Introduction

**Fig 1:**
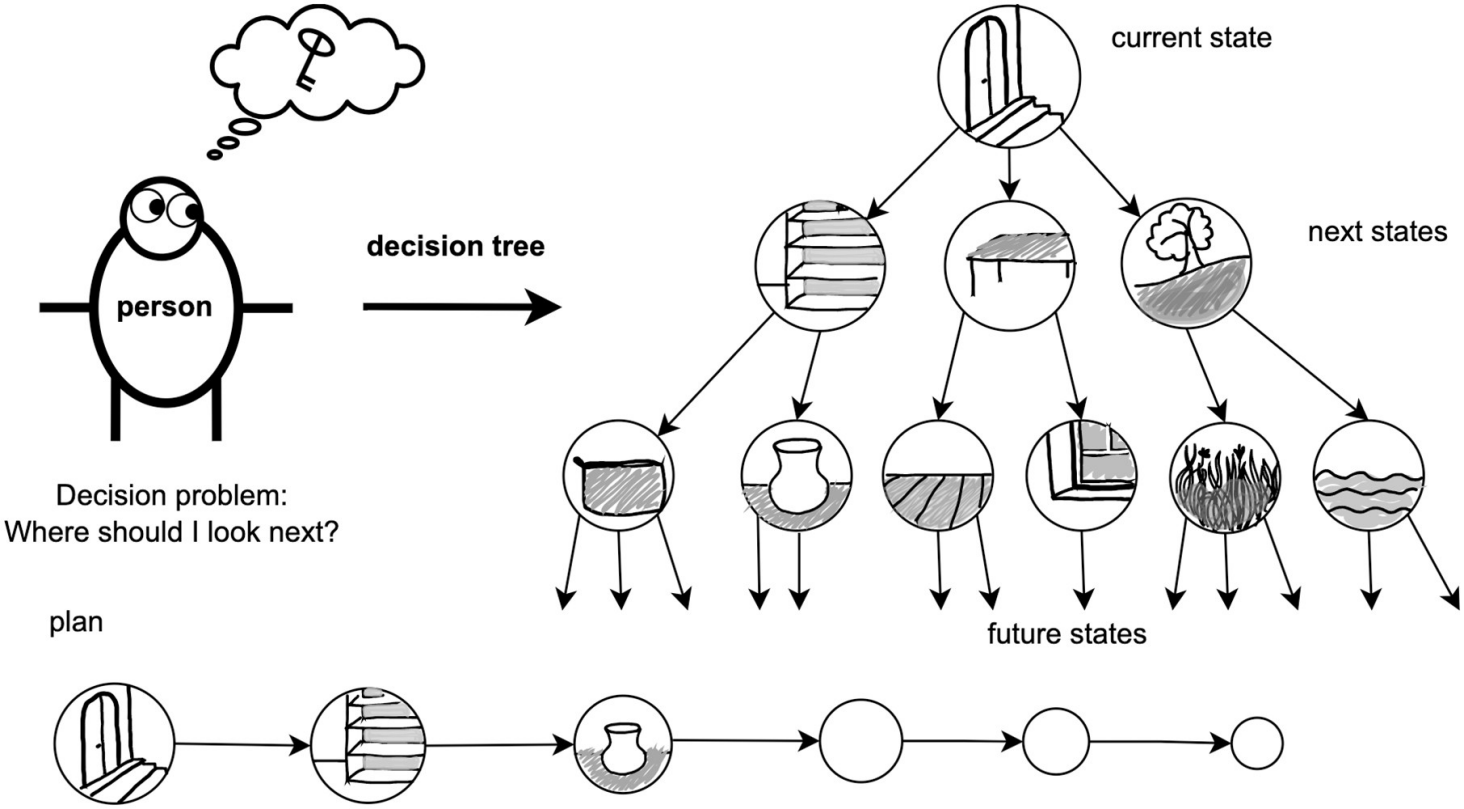
The decision problem facing an agent. The agent’s goal is to take actions that maximize long-run rewards. The agent can solve this problem by planning a path through a decision tree where nodes represent possible future states of the world, and edges represent actions the agent could take. The agent constructs the tree by iteratively considering possible future states, and plans by choosing the sequence of actions that minimizes costs, while taking into account the probabilities of success. Our task captures this process in a spatial setting, by mapping costs to steps taken to make an observation, and probabilities of success to the relative size of an observed area.

People make plans every day: working out a new route, playing a game, thinking through a possible conversation. Despite the ubiquity of planning, *how* people plan is an active area of research in psychology [1–4], economics [5], and computer science [6]. Planning involves making sequences of choices, where the possible actions that are available at each step depend on the outcome of the previous step. This process has been formalized as navigating a decision tree (Fig 1), which starts at an initial ‘root’ state, and continues until it reaches a ‘leaf’ that meets the goal criteria. For any situation beyond trivial toy problems, the growing complexity of a branching decision tree makes planning computationally costly. And yet, people daily face situations that require planning, and handle them remarkably well.

A prime example of a daily planning task that people are quite adept at is spatial planning. But while an enormous amount of work has studied and modeled planning in different domains (as we review below), to our knowledge relatively few studies considered detailed computational models of spatial planning in naturalistic contexts, under uncertainty. In this work, we take a step toward understanding human spatial planning under uncertainty using a novel Maze Search Task (MST), designed to resemble a natural environment where the costs are distances and rewards are spatial locations. This task simulates realistic contexts, such as navigation and search, where values intuitively derive from interactions between perception and cognition, while allowing to precisely measure probabilities and costs based on grid-world mechanics. A version of MST was previously used to study how people evaluate the goodness of plans made by others [7, 8], but a detailed computational account of how people themselves plan in the MST was not addressed. We use the MST to explore a family of cognitively-inspired computational planning models, that approximate expected utilities by integrating perceptual transformations, such as numerosity [9] and probability weighting [10]. We contrast these models both with an optimal Expected Utility model, and with a family of intuitive myopic heuristics, that could in principle be used to search an environment without planning ahead. In what follows, we briefly review recent relevant empirical work that attempted to model human planning. Based on this literature, we outline the relevant take-aways for the models we will consider in this work, including perceptual transformations and constraints that may influence how people plan in real-world.

First, while we mentioned that relatively little of the work on planning has focused on computational models of naturalistic spatial planning, certainly research has been done on it. For example, a small study modeled route planning within a city neighborhood as optimally solved by Breadth First Search [11]. However, larger empirical studies find that people rarely take shortest routes [12, 13], indicating that people likely plan using cognitive approximations when they navigate. Cognitive constraints that cause such approximations, or the mental computations implementing them, are not yet addressed in prior work.

Given the time constraints of in-lab studies, most prior work has focused on planning short action sequences in non-spatial situations, such as sequences of 2 or 3 actions, in rigorously designed yet simple experimental paradigms [2–4, 14, 15]. Several studies of games, such as Chess and Tic-tac-toe, have modeled planning over longer horizons in contexts with rich behavioral variation [16–19]. However, game state utilities in these tasks derive from task-specific heuristic game-board assessments that evaluate the player’s position based on various features of the board, making it hard to generalize the results beyond those specific games.

Several studies of planning suggest that people manage cognitive demands by limiting their planning horizon, which has implications for models of spatial planning. For example, when people are asked to connect moving consecutive disks to maximize their total volume, they do so in a way best explained as considering all possible paths up to a certain limited depth, in contrast to simple myopic heuristics, such as moving toward the largest disk [1]. In a different popular example, the Tower of London task requires stacking disks in a certain order, using the fewest moves. People doing this task increasingly deviate from the optimal number of moves as the depth of planning required to optimally solve the task increases, which again suggested that people use depth-limited planning [20]. In yet another example, several studies examined how people learn an implied decision tree in a three-stage bandit task, which involves making sequential decisions to learn and exploit a hidden task structure. People were found to prune their implied state-space during the choice stage in response to rising cognitive demands, suggesting that they relied on a flexible depth-limited representation to control cognitive costs [2, 4, 14]. Other examples of a limited depth of search are found in board games, such as Chess [17, 18, 21], and Four in a Row [16], where increasing expertise corresponds to greater depth in search algorithms [16–18, 21].

Given that several studies model limited planning horizon in various non-spatial tasks, prior literature provides strong evidence that limited planning horizon does occur under certain scenarios, and may generalize to realistic spatial contexts as well. Based on prior work, limited planning horizons can be implemented formally in several ways. Studies have limited planning depth by probabilistically pruning the planning tree at a each node, approximated by a discount rate applied to future rewards [2, 14], limiting the number of nodes explored by an algorithm, such as Monte Carlo Tree Search [16, 22], or planning up to a fixed depth [1]. Probabilistic tree pruning has been implemented as a discount rate, by assuming a mean-field approximation where at each step the future is weighted by the probability that it will be encountered (see Methods, [14]). Similarly, reinforcement learning routinely relies on discounting of future states to keep state-values from growing to infinity in tasks where horizon can be potentially unlimited [23], although this approach does not guarantee an optimal solution [24]. In this work, we implemented a limited planning horizon by discounting, which integrates naturally with our modeling approach (see below).

Beyond limited horizons, everyday planning often takes place in conditions of uncertainty, which may require gathering more information. In our task, uncertainty arises from the partial observability of the environment, as planning a search in MST means deciding in which order to observe a given maze. In the context of observing an unknown environment, people were previously found to use myopic strategies to choose the next observation [25]. Prior work in non-visual domains likewise tends to model information-seeking by myopic heuristics: choosing observations one at a time, rather than by planning ahead [26], with an extensive body of literature showing that people but tend to use cost-insensitive information search heuristics—that is consider the value of information, but not its cost [27, 28]. Though, in a recent exception, gathering information about costs and rewards in decision-trees of depths two and tree with deterministic state transitions was best explained by a family of models that go beyond single-step heuristics [3, 15, 29]. In [3, 15, 29] people saw a tree-like state-space structure, and had an opportunity to click on specific nodes to reveal their value for a given cost before navigating from the root to one of the leafs. The study found that during the initial information-seeking phase people optimized their total expected reward against the costs of gathering information, in contrast to heuristically selecting the next node (e.g. the closest node). They also found that people systematically used a variety of near-optimal planning strategies to reduce uncertainty and seek information relevant to achieving their goal, when information comes at different costs [29].

In addition to limited planning horizons and uncertainty, people’s plans rest on intuitive subjective utilities, which estimate the expected value of prospective states. Subjective utility functions can take different forms, examined in decision theories, such as Prospect Theory [10, 30, 31]. In real-world spatial planning, however, value derives from perceived physical quantities, such as number, and area. Given this, we hypothesized that real-world planning models may need to account for perceptual transformations over these quantities. In particular, the perception of numerosity is known to follow a non-linear relationship between the actual and perceived number [9], and probabilities tend to be perceived in a non-linear way that overestimates small and underestimate large probabilities. While probability perception has been widely explored [30–32], cognitive probability models are not yet integrated into studies of human planning. In our modeling approach we will consider previously found perceptual transformations of number and probability, and examine if they result in more human-like planning models.

Overall, previous computational and empirical work on human planning suggests that we need to build and evaluate planning models that have limited planning horizons and estimate intuitive utilities by using perceptual transformations. We also need to compare such models to simpler alternatives that do not involve planning ahead, which we broadly construe as myopic heuristics. Finally, we need a rich physically grounded environment to contrast the different models of planning in naturalistic settings. In the following sections, we detail the novel task we used to study people’s spatial planning, as well formally define planning models we compared people to.

## Materials and methods

### Maze search task

**Fig 2:**
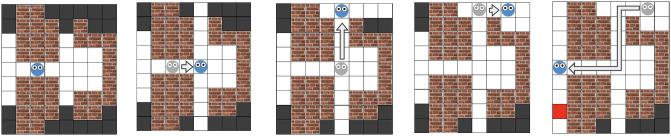
An example path in the Maze Search Task (MST). Black tiles are not-yet-observed areas, which hide an exit (red square). This maze has six ‘rooms’, groups of black tiles that are revealed all at once. Revealing tiles can be done in any order, but players are incentivized to plan their path so as to reach a hidden exit in fewer steps.

The objective of a participant in the Maze Search Task (MST) is to navigate a series of partially observable, two-dimensional grid-world mazes while minimizing the distance traveled to reach a hidden exit. Each maze consists of walls, corridors, and *rooms*—clusters of hidden tiles that can be observed together. One of the tiles contains an exit, which remains hidden until the room containing it is observed. Participants are told that each of the black tiles is *equally likely* to hide the exit, and are instructed to reach the exit in as few steps as possible. The exit becomes visible as a red grid tile once its location is observed. Fig 2 shows a maze with the player’s location indicated by the face avatar, and the player’s path indicated by arrows. An experiment demo is available at https://marta-kryven.github.io/experiments.html. The complete task instructions are available in Supplementary Materials S6 Appendix.

**Fig 3:**
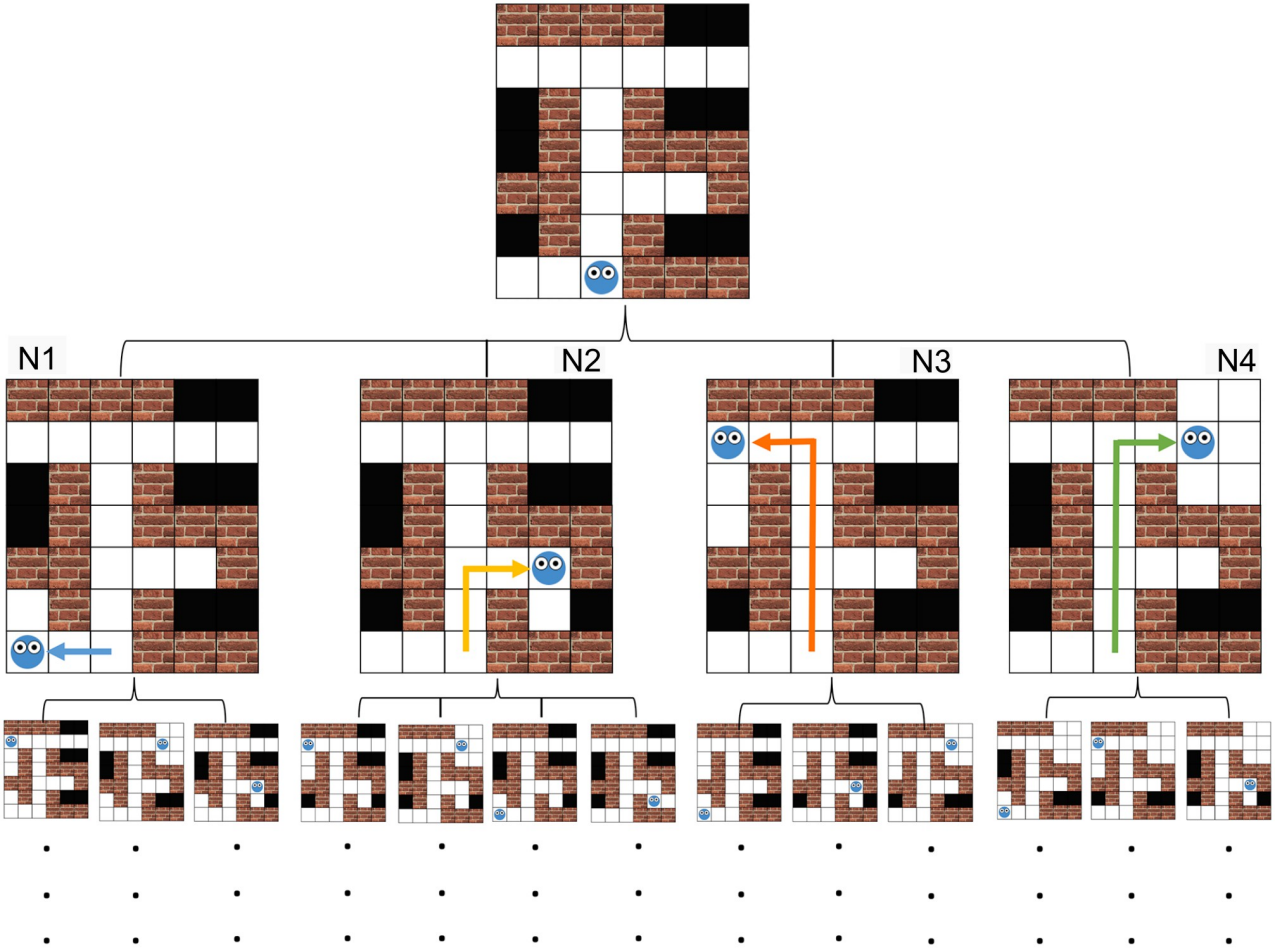
Decision-tree for a maze with four rooms (hidden tiles that are revealed together). The tree abstracts away from specific moves like ‘up’ and ‘left’ and considers more general actions like which area to uncover next. The root of the decision-tree corresponds to the player’s starting location. The four nodes accessible from the root indicate the possible observations that can be made next, followed by the observations that can follow each of those, and so on.

The player in the MST can move to any adjacent grid tiles that are not blocked by walls in the four cardinal directions (up, down, left, right), and reveal the black unobserved tiles by bringing them into the avatar’s line of sight, or isovist. To compute the agent’s isovist in the context of a grid-world, we consider a cell to be visible from the agent’s location if its entire area can be seen from anywhere within the agent’s current cell, taking into account any obstacles. For example, the diagonal cell at node N2 in Fig 3 is not visible, as it’s upper-right corner can not be seen from anywhere inside the agent’s cell. Upon reaching the exit the player is moved to the next maze. If the current maze is the last trial in the experiment, then upon reaching the exit the experiment ends.

### Computational models

To plan a route through a maze, we first define a decision-tree detailing all possible orderings in which the rooms in a maze can be revealed. Each node in this tree corresponds to a unique combination of a location (*x*, *y*) from which an observation is made, and the list of all observations so far, including the newly observed area. If a room can be observed from two different locations, then this room will be represented by two nodes in the decision tree, each corresponding to a unique location from which an observation is made. An example of a maze along with a partial decision tree for this maze is shown in Fig 3. The starting location corresponds to the root of the decision-tree. The leaves correspond to all possible unique ordering of observations through which the entire maze can be revealed.

Planning a path through a maze entails computing costs of decision tree nodes *C*_*k*_, and mapping the node costs to probabilities of choosing them. We use a softmax mapping, a standard method of modeling expressed preferences in decision-making [2, 33–36]:

$$\begin{array}{c} \sigma {\left(C\right)}_{k}=\frac{\text{exp}(-C_{k}/\tau )}{\sum_{j}\;\text{exp}\left(-C_{j}/\tau \right)} \end{array}$$ (1)

Here, *τ* is the temperature parameter that controls the strength of the softmax mapping, *k* indexes tree nodes, and *j* indexes siblings of node *N*_*k*_. The negative sign in front of the cost ensures that shorter paths result in higher probabilities. As *τ* → 0, the agent will always choose the shortest expected path. As tau increases, the agent will behave in a more noisy way, and as *τ* → ∞ agents will chose actions at random.

#### Planning ahead

We first define the optimal Expected Utility cost function, which computes the shortest paths to the exit. We will use the optimal model to derive other, approximate planners.

#### The Expected Utility (EU)

model defines the cost of a node *N*_*i*_ as given by the expected number of steps to the exit if this node is chosen, assuming all subsequent choices are optimal as well:

$$\begin{array}{c} C_{EU}\left(N_{i}\right)=s_{i}+p_{i}e_{i}+\left(1-p_{i}\right)\underset{c_{j}\in Child\left(N_{i}\right)}{\text{min}}C_{EU}\left(c_{j}\right). \end{array}$$ (2)

Where *p*_*i*_ is the probability that the exit is found at *N*_*i*_. Assuming the exit is equally likely to be in any of the black tiles, *p*_*i*_ is the ratio of the number of tiles observed at *N*_*i*_ to the total number of unobserved tiles remaining in the maze. In a general case this probability could be arbitrary. Further, *s*_*i*_ is the number of steps to reach *N*_*i*_ from its parent node in the tree—that is, the number of steps between the previous observation, and the observation at *N*_*i*_; *e*_*i*_ is the expected number of steps to the exit from *N*_*i*_, if the exit is observed at *N*_*i*_—in other words, the average number of steps to a cell revealed in *N*_*i*_. Lastly, *Child*(*N*_*i*_) is the set of children of *N*_*i*_ in the planning tree—that is, a set of observations that can be made next, after reaching *N*_*i*_.

#### The Discounted Utility (DU)

model modifies the EU model by discounting the costs of future nodes, by a rate of *γ* ∈ [0, 1], to implement a limited planning horizon:

$$\begin{array}{c} C_{DU}\left(N_{i}\right)=s_{i}+p_{i}e_{i}+\gamma \left(1-p_{i}\right)\underset{c_{j}\in Child\left(N_{i}\right)}{\text{min}}C_{DU}\left(c_{j}\right) \end{array}$$ (3)

#### The Probability Weighed Utility (PW)

model modifies the EU model by transforming probabilities, using a weighting function of the form *π*(*p*) = *exp*(−|ln(*p*)|^*β*^) [10]. While probability weighting is widely used to model probability perception in monetary gambles [30–32] it is not commonly evaluated in the context of planning.

We define the PW cost function as follows:

$$\begin{array}{c} C_{PW}\left(N_{i}\right)=s_{i}+\pi \left(p_{i}\right)e_{i}+\pi \left(1-p_{i}\right)\underset{c_{j}\in Child\left(N_{i}\right)}{\text{min}}C_{PW}\left(c_{j}\right) \end{array}$$ (4)

This probability weighting function has an effect of overestimating small probabilities and underestimate large probabilities when 0 < = *β* < 1, and has the opposite effect when *β* > 1. If *β* = 0 all probabilities have the same uniform value, and *β* = 1 is equivalent to the optimal Expected Utility in which all probabilities are equally weighted.

#### The Probability Weighting and Discounting (PW-DU)

model combines probability weighting and discounting in a model with three free parameters—*τ*, *γ*, and *β* (corresponding to the softmax temperature, the discount rate, and the probability weighting respectively).

$$\begin{array}{c} C_{pw-du}\left(N_{i}\right)=s_{i}+\pi \left(p_{i}\right)e_{i}+\gamma \pi \left(1-p_{i}\right)\underset{c_{j}\in Child\left(N_{i}\right)}{\text{min}}C_{pw-du}\left(c_{j}\right) \end{array}$$ (5)

#### Expected Utility with Numerosity psychophysics (EU-Num)

When people reason about varying amounts of hidden tiles, they could in principle count the tiles exactly. At the same time, people could instead roughly estimate the number of tiles, causing systematic deviations [9, 37]. We use a recent information-theoretic numerosity model to account for the latter possibility [9]. The information-theoretic numerosity model has one free parameter, bit threshold *B*—the number of bits processed by the perceptual system to estimate numbers, such that people who count the tiles can be modeled with a large *B*, and people who guess at a glance can be modeled with a small *B* (we used *B* ∈ [0.1, 10]).

To fit bit threshold *B* to people, we generate a table of mappings between observed quantities *n* ∈ [1, 80] (an empirical upper bound on the number of unobserved cells in a maze) and an expectation of a subjective estimate *q*, assuming different levels of *B* ∈ [0.1, 10]. The upper bound on *B* is empirically chosen as sufficient to perceive 15 cells exactly, which we deem sufficient, given that human subitizing range is shown to be limited to about 6 [38]. The lower bound is an empirically chosen minimum with a subitizing range below 2. We use this table to transform numbers of tiles used when computing node costs in the models parameterized with numerosity, substituting *q* for *n* given each level of *B*. This transformation affects the model’s estimates of the number of steps between rooms, room sizes (the number of tiles that would be revealed by an action), and the total number of remaining tiles. Perceptual number transformation also has an indirect effect on probability perception, as it affects both the number of cells in a room and the total number of cells in a maze, in a non-linear way that depends on B. See Supplementary Materials S2 Appendix for more details. To estimate the expectation, we computing the probability distribution *Q*(*k*|*n*, *B*) that an observed numerosity *n* is represented by *q* using equation (5) from [9]. We set a prior over numbers as probability of how often a numerosity *n* is encountered, *P*(*n*) ∝ 1/*n*2 according to Zipf law, following the approach used in [9].

#### Discounted Utility with Numerosity psychophysics (DU-Num)

The DU-Num incorporates information-theoretic numerosity into the DU model along the lines above.

#### Monte Carlo Tree Search Sampling model (Sampling)

The planning models considered so far accurately represent the planning tree, but assume that the utilities of its nodes are approximated in some way. Another way to make approximate plans is to construct a partial planning tree. This process can be formalized by the Monte Carlo Tree Search (MCTS), an algorithmic framework for simulating multiple possible outcomes while keeping track of them in a tree, and choosing the best one based on the simulation results. The accuracy of the planning tree in MCTS is controlled by two free parameters—the computational budget that controls how many nodes are sampled, and exploration that controls how greedy or stochastic the process is [39]. Implementations of MCTS have been previously used to model games such as chess, go, and four-in-a-row [16]. Here we implement MSTC to approximate the EU model, with it does with increasing accuracy as the budget parameter is increased. We point the interested reader to Supplementary Materials S1 and S2 Appendices for the details of this implementation, and for an alternative formulation of the Maze Search problem using a classic reinforcement learning notation for Partially Observable Markov Decision processes in a grid-based state-space.

#### Myopic heuristics

A large body of decision-making literature has focused on heuristic solutions to problems, where a heuristic derives from simple rules or features, such as the size of an area [1, 26, 40]. While it is impossible to enumerate all the possible heuristics that could be invented in response to a given situation, we considered 7 one-step heuristics based on prominent features of MST, to rule out simple interpretations of the task.

#### Cells heuristic (Cells)

The cost of a node is taken to be the number of tiles observed at that node: *C*(*N*_*i*_) = −*cells*_*i*_. We use a negative sign because revealing more cells results in a *smaller* expected cost for finding the exit. This approach greedily minimizes the entropy over goal locations, similar to previously studied cost-insensitive information search heuristics, e.g. [27, 28].

#### Steps heuristic (Steps)

The cost of a node is taken to be the number of steps to the node from its parent: *C*(*N*_*i*_) = *s*_*i*_. This approach treats the cost of an observation as reduced to its immediate cost, disregarding future states to which this observation might lead.

#### Steps-Cells heuristic (S-C)

A combination of the two heuristics above. The cost of a node is a combination of the steps to get to it and the revealed cells, but without planning more than one step ahead: *C*(*N*_*i*_) = *ks*_*i*_ − (1 − *k*)*cells*_*i*_, where *k* ∈ [0, 1] is a free parameter. This heuristic balances value of information against its costs, similarly to one of the models explored in [27].

Adding information-theoretic numerosity to each of the three heuristics results in three more modified heuristic models—Steps with numerosity **Steps-Num**, Cells with numerosity **Cells-Num**, Steps-Cells with numerosity **Steps-Cells-Num**. Lastly, we consider a **Random** policy, in which the value of any node is the same, *C*(*N*_*i*_) = 1.

#### Different paths preferred by different models

**Fig 4:**
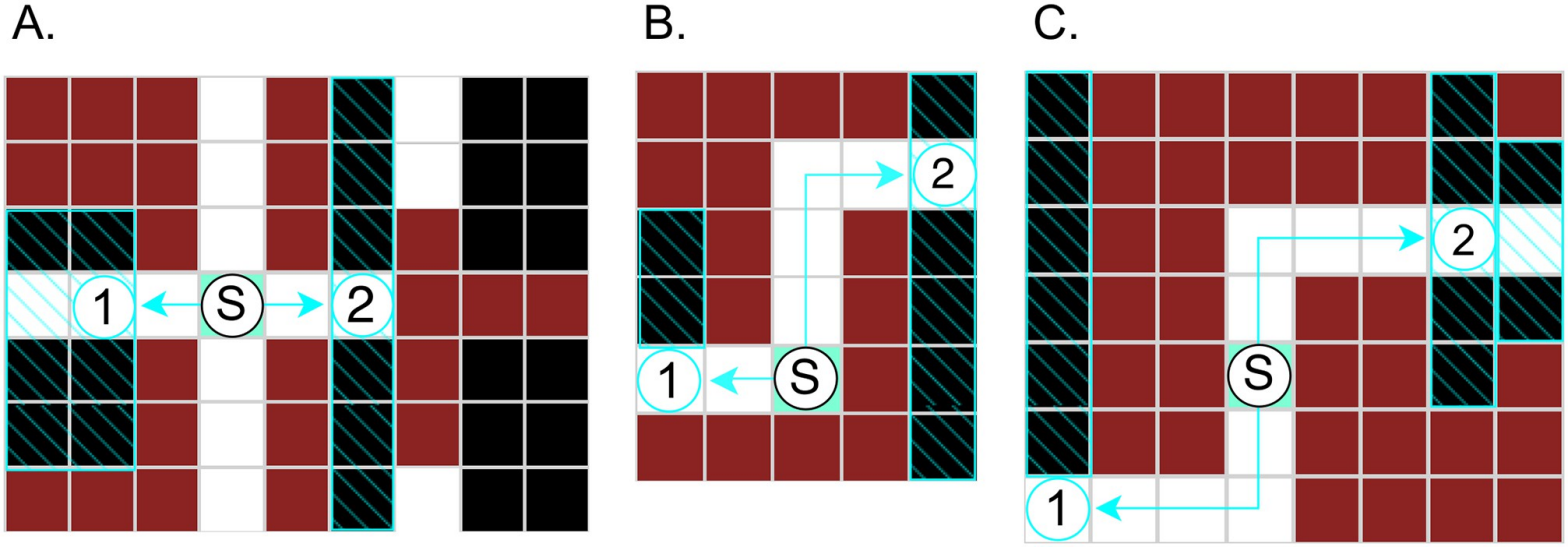
Examples of maze designs illustrating differences between models’ predictions. Here “S” indicates the starting position (the root of the decision tree). The initial observations accessible from the starting location are numbered as 1 and 2. Hatching indicates cells that will be revealed by traveling in each direction. **A.** In the initial decision heuristics can not distinguish between the available choices, as both nodes can be reached in 2 steps, and reveal 6 cells each. **B.** The Expected Utility model is indifferent between the two directions, as it trades probabilities of success in each direction against the distance cost. In contrast, the Discounted Utility model, and the Steps heuristic can both predict the human preference for visiting the closer room. **C.** In this example all models except the Discounted Utility model are indifferent between the two directions. The Discounted Utility model can predict human preference for going right (node 2).

The models described above can make different predictions about how a maze should be traversed, which allows us to differentiate between planning or heuristic solutions, and between individual planning models. Fig 4, shows three simple illustrations of how maze designs can bring out such differences. See Supplementary Materials S3 Appendix for more examples, along with simulations plots showing how the probability of choosing each direction depends on model parameters.

At the simplest level, the Steps heuristic predicts a preference for closer rooms, regardless of their shape or size. Since the Steps heuristic makes this choice one step at a time, the path it takes to search an entire maze does not minimize overall steps. The Cells heuristic can explain a preference to open larger rooms, one step at a time, regardless of distance.

Fig 4B shows a maze where the EU model is indifferent between the two directions, but the heuristics have a clear preference. Here, the Steps heuristic prefers the closer room, and the Cells heuristic prefers the bigger room. The Steps-Cells heuristic combines the Steps and Cells within a single one-step rule, but will still be indifferent between choosing one of two equidistant rooms with the same number of cells, even when they lead to different opportunities on the next step, as for example, shown in Fig 4A.

The DU model can prioritize accumulating observations earlier in the path, even if the overall distance traveled remains the same or increases. This becomes apparent even in simple scenarios such as shown in Fig 4B and Fig 4C, where the EU model is indifferent between the two directions, but the DU model has a clear preference for one of the rooms, as it can discount the possibility of having to visit the other room next. In Fig 4B both the DU and the PW model can predict the human preference for visiting the closer room first. The PW model can do this by relaxing the disparity in room sizes, and the DU model does this by discounting the path length leading toward the larger room on the second step. Fig 4C shows a maze where neither the optimal EU model nor heuristics can differentiate between the two directions, as both reveal rooms of an equal size that are equidistant from the observer, but the rooms differ in the average distance to a cell that will open. In this example, the majority of people search the triangular-shaped room first (shorter average distance to a revealed cell), and the DU model accurately predicts this preference. While all planning models take into account the average distance to cell in a room that will open, only discounting makes the path that delays visiting the room with the longer average distance to a revealed cell less costly.

While probability weighting principle in model PW has been previously explored to model how people choose between gambles, extending it to sequential choices in the spatial search context has unique implications. First, for *β* < 1 PW makes room of different size seem more alike, so that for all cases where the optimal model EU takes a longer path toward revealing more cells, there exists a *β* < 1 that reverses this preference. This has an effect of biasing the planning to place more weight on distance. Second, values of *β* > 1 make rooms of different sizes appear to be more different, such that the planning puts more weight on probabilities. Boundary values of *β* = 0.1 will guide the model to behave like a variant of the Steps heuristic with look-ahead—that is, minimizing the length of the overall path to reveal the entire maze, rather than minimizing the length of path to reach the exit. Boundary value of *β* → ∞ will behave like the Cells heuristic with look-ahead. See Supplementary Materials S2 Appendix for more details, including examples of how model PW can in principle traverses a large maze in two different ways depending on its parameters, and do so by a trajectory distinctly different from the optimal EU model. In other words, the space of models we consider spans a wide range of possible behaviors, due to very different commitments they make about how one plans through a spatial environment.

### Data analysis methods

#### Model fitting

The models were fitted at the individual level by considering all decisions made by an individual during the experiment, which are treated as independent of each other. A ‘decision’ in our case refers to choosing one of two or more child nodes, meaning choosing which one of the unobserved rooms to search next.

#### Model performance as likelihood

We fit our models to individuals, and obtained out-of-sample predictions using fivefold cross-validation. The model fitting procedure sets a prior on softmax temperature *τ* inversely proportional to its magnitude, but assumes a uniform prior over all other parameters (e.g. *γ*, *B*, *β*, *k*). We measured the *model performance* as the total test log likelihood (LL) of the model across all five test folds. This metric accounts for the flexibility of the different models without parameter counting, in contrast to AIC and BIC which can be a poor measure of flexibility [41]. Differences in this cross-validated LL (ΔLL) can be interpreted similarly to differences in AIC: ΔLL = 1 is roughly equivalent to ΔAIC = 2. The Supplementary Materials S4 and S5 Appendices also show model performance analyzed using Monte-Carlo cross-validation. We analyzed *individual differences in planning* by comparing the likelihood of the best fitting planning model (one of EU, EU-Num, PW, DU, DU-Num, PW-DU, Sampling) and best fitting heuristic (one of Steps, Cells, Steps-Cells, Random, Steps-Num, Cells-Num, Steps-Cells-Num) for each individual.

#### Model performance as correlation

We also considered a model’s ability to explain *behavior aggregated across participants*. To do this, we computed the correlations between model predictions, and the probability that people will make the corresponding choice in the following way. We first computed the probabilities of participants making each choice at the initial decision point in each maze, aggregated across individuals. Individuals search each maze in different ways (with each person potentially facing a unique set of choices), but the initial decisions are shared by all participants in the experiment. We then correlated these probabilities with each model’s predictions, where the models are parameterized with the mean parameters of the experimental population. While we consider the first choice to be the most indicative and carefully controlled measure, in Supplementary Materials S4 and S5 Appendices we also show correlations computed using all decisions in the experiment that were visited by at least 20% of the participants.

#### Decision time analysis

There are two types of decision times in Maze Search:

(1) **Initial decision time** to make the first move at the start of the trial, during which people form a mental representation of a maze, and plan a search within a certain planning horizon (or choose where to observe next using a step-wise heuristic)—the time at the root of the decision tree.
(2) **Subsequent decision time**, corresponding to inner nodes of the decision tree where people either make a pre-planned move, or decide where to observe next using a step-wise heuristic. We analyze both types of decision times to assess the relationship between the model parameters fitted to people and the extent of mental computations, as evidenced by decision times.

The simplest interpretation of decision times is that they reflect precision of computation, so that longer Initial decision times should indicate more accurate planning. Additionally, more planning may lead to Shorter subsequent decision times, as people making a per-planned move should need less time compared to people who compute a step-wise heuristic. Without making specific assumption about implementation, precision of computation is measured by the fitted softmax temperature *τ* of the optimal EU model, (a smaller *τ* is more optimal). To test whether decision times reflect precision of computation, we fit a linear regression model predicting decision times by fitted softmax temperature in the EU model. Since a smaller *τ* corresponds to closer approximation of expected utility, by our hypothesis we would expect to see a negative slope *α* for Initial decision times, and a positive slope for Subsequent decision times.

Decision times may also reflect people’s planning horizon, as measured by fitted discounting parameter *γ*, in which case a higher *γ* (more planning, less discounting) fitted to people should corresponds to longer Initial and shorter Subsequent decision times. To test this hypothesis, we compute linear regression predicting decision times from discounting parameters fitted to people in models DU, DU-Num, and PW-DU. Since a higher *γ* corresponds to more planning and less discounting, by our hypothesis we should see a positive slope at *γ* for Initial decision times, and a negative slope for Subsequent decision times.

### Experimental procedure

The experiment was conducted in a web browser, using a JavaScript and PHP interface developed by the authors. Participants first read a consent page and a short description of the Maze Search Task. Following consent, participants read a detailed description of the task, completed maze navigation practice, and answered an instruction quiz. The quiz included questions about the objectives of the task, task controls, and the line-of-sight mechanism by which the mazes are revealed. Participants could not proceed to the experiment until they submitted the correct answers to the quiz. On each trial a participant was placed at the starting position, and navigated by clicking on one of the adjacent tiles, until the exit was reached. The starting locations in each maze were predetermined, and the exit locations were randomly chosen for each maze at the time of design.

After completing the experiment, each participant answered a demographic questionnaire, and provided a free-form description of search strategies they used in the experiment. Participants were paid a US minimal wage and received a performance-based bonus, configured so that on average 70% of individuals receive a bonus. Experiments were approved by the Institutional Review Board at Massachusetts Institute of Technology. Participants gave written consent. The same procedure was used for both experiments. Please see Supplementary Materials S6 Appendix for an explanation of the bonus scheme and task instructions.

## Experiments

### Experiment 1

In the first experiment we aimed to test whether people’s planning follows optimal expected utility. The experiment included 40 mazes, presented in random order. Of the mazes, 23 were two-room mazes corresponding to simple decision trees with two leafs. The two-choice mazes were designed to be similar to the experimental paradigms that involve choosing between two gambles, traditionally used to study subjective utilities [30, 31]. They were designed so that the bigger of the two rooms also requires more steps to reach than the smaller room, to bring out differences between human choices, and the EU model. In terms of Expected Utility of these binary choice mazes, they were designed so that:

(1) The Expected Utility is not predicted by differences in distance or size alone, and
(2) The Expected Utility of the bigger and further room is larger or equal to that of the closer and smaller room.

This was done because in the pilot experiments we noticed that people prefer closer rooms more often than the EU model. Another 4 mazes consisted of 2 rooms, but included a looped corridor, meaning that the decision tree was not strictly binary. The remaining mazes consisted of 3–4 rooms. See Supplementary Materials S4 Appendix for full set of mazes used in the experiment, and additional details.

We recruited 120 US participants on Amazon Mechanical Turk, of which 4 were excluded for failing to answer instruction quiz correctly in two attempts. In total 116 participants (56 female, 60 male, *M*(*age*) = 39, *SD*(*age*) = 12) were included in the analysis. Rerunning the analysis with all participants included did not change the results. On average the experiment took 14 minutes to complete, with people making on average 51.5 decisions during this time.

Given the lack of comparable studies there was no immediate way to establish the requisite participant number for a correct power analysis. Pilot studies showed that 100 participants were more than sufficient to show variability between people, and so 120 participants was erring on the side of caution.

#### Results

#### People do not plan according to optimal expected utility

**Fig 5:**
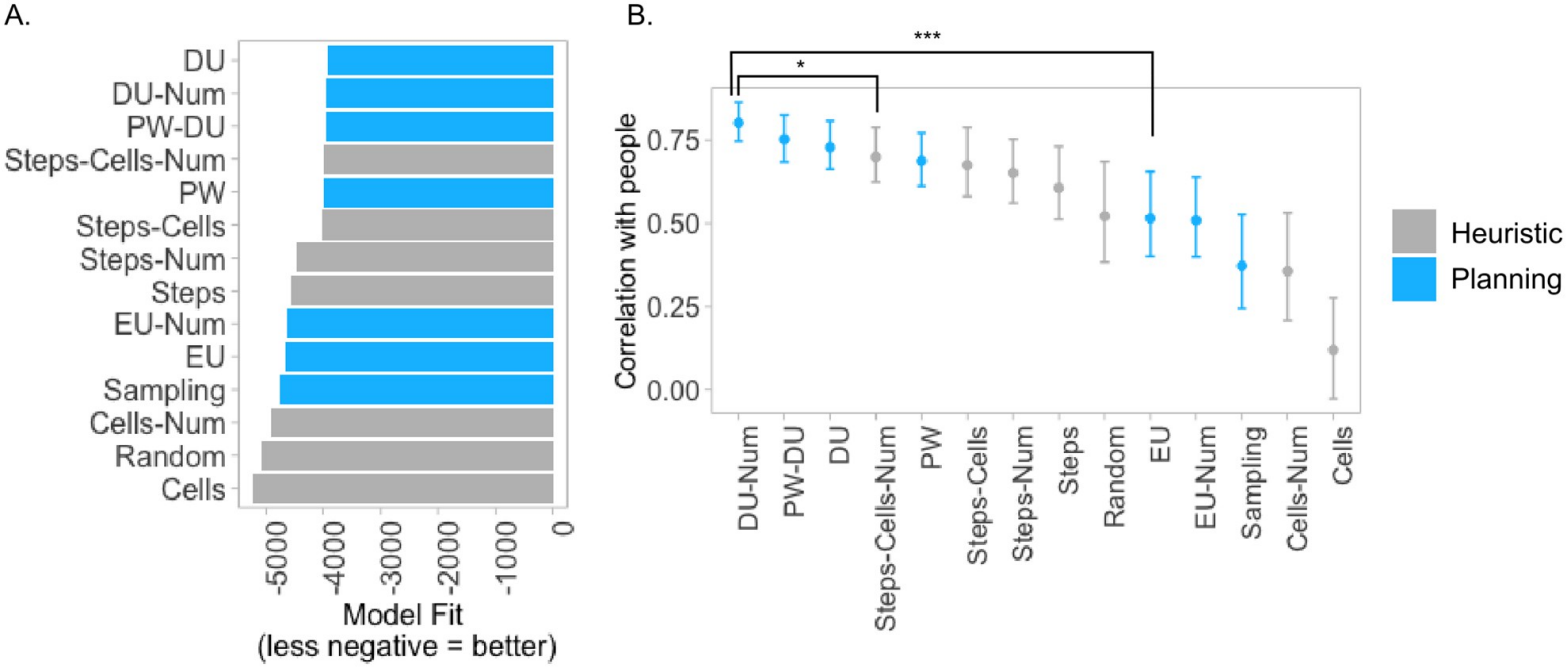
Experiment 1, results. **A.** Model performances, measured as the total log likelihood of each model across all five folds. Shorter bars indicate better fit to human behavior. **B.** Bootstrapped correlations of choice probabilities aggregated across participants with each model’s predictions. Error bars indicate 95% confidence intervals.

Model performance is shown in Fig 5A, with the models ordered by their likelihood. The difference in log-likelihood between EU and the best most likely planner DU is Δ*LL* = 764. Fig 5B shows the bootstrapped correlations between the probabilities of decisions made by people and the models. Models were parameterized with the mean parameter values fitted to human distribution, shown in Supplementary Materials S4 Appendix. The correlation of the best-performing DU-Num model with people is *r* = .81, (95*CI*[.74 − .91]), and the correlation of EU is *r* = .49, (95*CI*[.31 − .71]). These correlations are significantly different, with bootstrapped difference between their means of [.1 − .5], indicating that the DU-Num model predicts the aggregate population better than the optimal EU model. Graded correlations of each model with peoples’ choices are shown in Supplementary Materials S4 Appendix. Overall, we find that while several computational models can reasonably predict human planning, all of them plan ahead in a way that deviates from the model based on optimal expected utility.

#### Evidence in favor of planning over heuristics

We also found that planning models are better at explaining people’s behavior, compared to myopic heuristics. The difference in log-likelihood between the most likely planner DU and the most likely heuristic Steps-Cells-Num is Δ*LL* = 47. Bootstrapped correlations between models and people’s choices in Fig 5B show that the 95% CI of bootstrapped difference between correlations of DU-Num (a planner with the highest correlation) and Steps-Cells-Num (a heuristic with the highest correlation) is significant, [.01 − .2], suggesting that people’s choices are best explained by models that plan. See Supplementary Materials S4 Appendix for more analysis, including variability between individuals.

#### Depth-limited planning

Fig 4C shows a maze from Experiment 1 where the DU model can explain human preferences, but other models can not. Here, the DU model prefers searching room ‘2’ first, while the other models are indifferent between ‘1’ and ‘2’. This environment has occurred in Experiment 1 twice, in the original, and in a rotated form (see Supplementary Materials S4 Appendix for the full list of mazes). In both presentations, the proportion of people who searched the triangular room first was significantly greater than chance (0.71, *χ*^2^ = 19.6, *df* = 1, *p* < .0001 and 0.68, *χ*^2^ = 14.3, *df* = 1, *p* < .0001). The DU model is able to distinguish between the ‘long’ room, in which in the worst case scenario the exit may be still 6 steps away when the room is opened, and the ‘compact’ room, in which the exit will be at most 2 steps away. The EU and PW models can not distinguish between these two directions, as the exit is equally likely to be in either room. Distance and size based step-wise heuristics do not distinguish between these two directions, as both rooms require 5 steps to reach, and both rooms contain 6 unrevealed tiles.

In summary, Experiment 1 shows that people deviate from the optimal Expected Utility in spatial settings, and demonstrates that only a discount rate can explain this deviation in critical scenarios. At the same time, while the difference between approximate planning models and heuristics is significant, the differences are small, due to the experimental design dominated by simple two room mazes. In the next experiment we aimed to further differentiate between planning and heuristics, by focusing maze design on decisions that elicit planning.

### Experiment 2

In the second experiment, we aimed to differentiate between models that plan ahead and myopic heuristics. We followed the experimental procedure from Experiment 1 with a new set of 23 mazes containing between 4 to 10 rooms. The mazes were designed so that in the initial decision in each maze heuristics could not distinguish between the available choices, but planning models could. Supplementary Materials S5 Appendix shows the full set of mazes used in the experiment, illustrating this design. Since each direction of travel during an initial decisions leads to a room of the same size after taking the same number of steps, neither direction is preferable to a heuristic. However, the areas of a maze that become accessible in subsequent decisions differ between directions, meaning that planning models prefer one of the directions over the others.

We recruited 107 US participants on Amazon Mechanical Turk, of which 7 were excluded for failing to answer instruction quiz correctly in two attempts. In total 100 participants (35 female, 80 male, *M*(*age*) = 33, *SD*(*age*) = 9) were included in the analysis. Rerunning the analysis with all 107 participants included did not change the results. The experiment took on average 20 minutes to complete, with people making on average 70 decisions during the experiment.

#### Results

#### Evidence in favor of planning over heuristics

**Fig 6:**
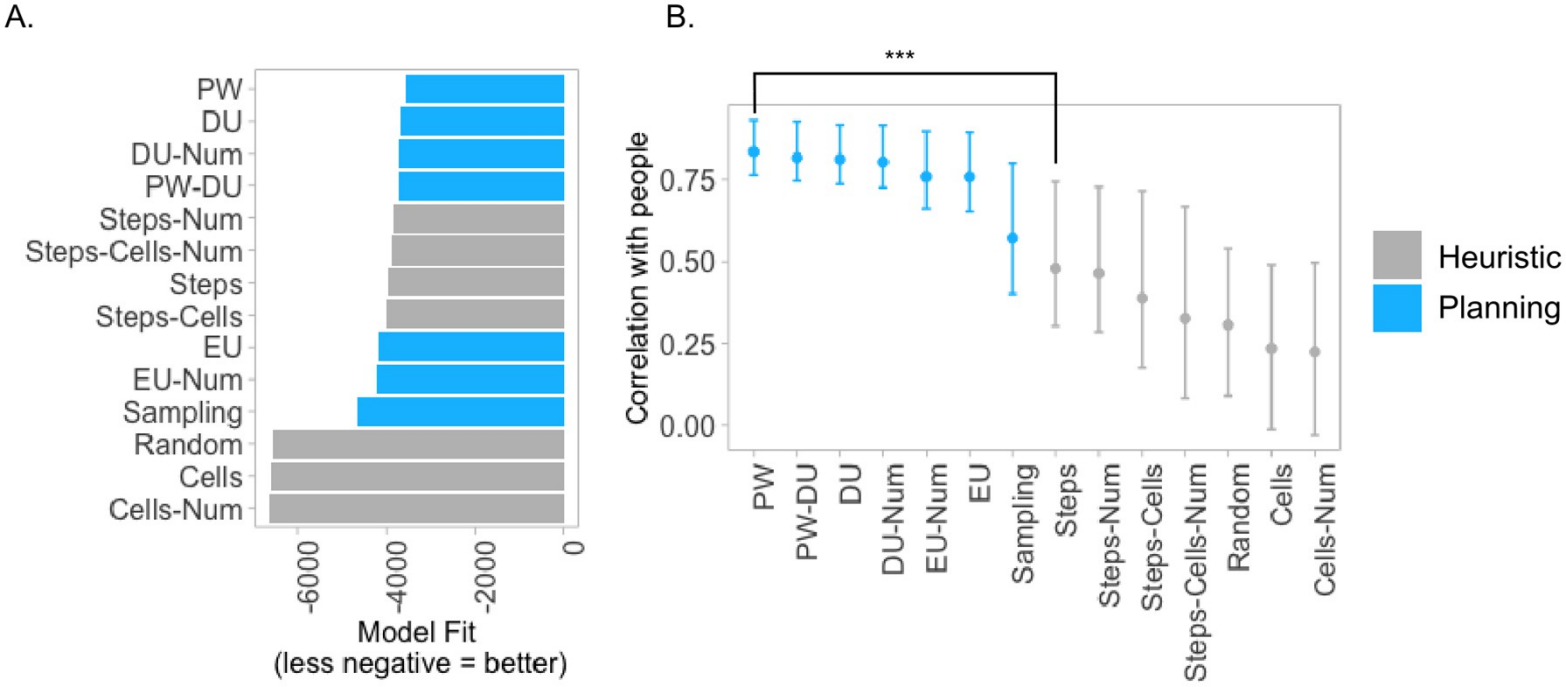
Experiment 2 results. **A.** Model performances, measured as the total log likelihood of each model across all five folds. Shorter bars indicate better fit to human behavior. **B.** Bootstrapped correlations of choices aggregated across participants with each model’s predictions. Error bars indicate 95% confidence intervals.

Overall, we found that planning models are better at explaining people’s behavior compared to myopic heuristics. Fig 6A shows model performance with the models ordered by their likelihood. The difference in likelihoods between PW and the best-fitting Steps-Num heuristic is Δ*LL* = 262. The 95% CI of bootstrapped difference between correlations of PW (a planner with the highest correlation) and Steps (a heuristic with the highest correlation) is [0.2 − 0.5], as shown in Fig 6B, indicating that planning models predict the aggregate population better than myopic heuristics. See Supplementary Materials S5 Appendix for more analysis, including variability between individuals, graded correlations of each model with peoples’ choices, and the distributions of model parameter values fitted to individuals.

#### Depth-limited planning

Below we show that our modeling approach can predict human decision times. First, we test whether decision times reflect precision of computation, by fitting a linear regression model *time*_*i*_ = *α*_0_ + *α*_1_*τ*_*i*_ + *e*. Here *α*_0_ is the Intercept measured in milliseconds, *i* indexes people, *τ*_*i*_ ∈ [0, 10] is the temperature parameter fitted to people in the EU model, and *time*_*i*_ is decision time. Since a smaller *τ* corresponds to closer approximation of expected utility, for our hypothesis to hold, we expect to see a negative slope at *τ*_*i*_ for Initial decision times, and a positive slope for Subsequent decision times. We found the slopes to be significant in line with this expectation, indicating that *τ* reflects the extent of planing in maze search (see Table 1). Supplementary Materials S5 Appendix shows data used to compute the model in Table 1).

**Table 1:** Linear regressions predicting decision times by parameters fitted by model EU.

Times	*p*	F-statistic	*α*_0_	*α*_1_ (*τ*)
Initial	< .0001	F(1,2322)=32	3515	-255
Subsequent	< .0001	F(1,6045)=29	905	55

Next, we test whether human decision times can be explained by limited planning depth, as measured by the discounting parameter *γ* ∈ [0, 1] fitted to people. We compute linear regression of the form *time*_*i*_ = *α*_0_ + *α*_1_*τ*_*i*_ + *α*_2_*γ*_*i*_ + *e*, where *γ*_*i*_ and *τ*_*i*_ are fitted to people in model DU. Since a higher *γ* corresponds to more planning and less discounting, by our hypothesis we expect to see a positive slope at *γ*_*i*_ for Initial decision times, and a negative slope for Subsequent decision times. We found the regression slopes to be significant and in line with this expectation, as shown in Table 2, providing further evidence in support of decision times explained by limited planning depth.

**Table 2:** Linear regressions predicting decision times by parameters fitted by model DU.

Times	*p*	F-statistic	*α*_0_	*α*_1_ (*τ*)	*α*_2_ (*γ*)
Initial	.0001	F(2,2321)=9	2880	−163	530
				*p* = .03	*p* < .0001
Subsequent	< .0001	F(2,6044)=10.4	1069	16	-134
				*p* = .3	*p* = .001

A similar regression models for parameters fitted to people in model DU-Num further support our conclusion. For model DU-Num, regression model takes the form *time*_*i*_ = *α*_0_ + *α*_1_*τ*_*i*_ + *α*_2_*γ*_*i*_ + *α*_3_*B*_*i*_ ++ *e*, where *B*_*i*_ ∈ [0, 10] is the threshold of bits processed when estimating the numbers of tiles (larger *B*—more accurate). The results of the fitted regression model in Table 3 show slope coefficients for *γ* to be significant, supporting the hypothesis that decision times depend on planning depth. We also observe a positive slope associated with the information-theoretic numerosity parameter *B*, meaning that higher value of *B* is associated with longer deliberation times in both types of decisions. This result provides further evidence in support of our modeling approach, as it associates longer decision times with more accurate number perception.

**Table 3:** Linear regressions predicting decision times by parameters fitted by model DU-Num.

Times	*p*	F-statistic	*α*_0_	*α*_1_ (*τ*)	*α*_2_ (*γ*)	*α*_3_ (*B*)
Initial	< .0001	F(3,2320)=20	2612	-340	607	88,
				*p* = .02	*p* < .0001	*p* < .0001
Subsequent	< .0001	F(3,6043)= 39	951	76	-192	26.6
				*p* = .03	*p* < .0001	*p* < .0001

In the PW-DU model probabilities are perceived most accurately when *β* = 1, while any deviation from this value leads to less exact perception of probabilities, meaning that the relationship between *β* and probability perception is non-linear. Therefore, one way to compute a regression model for parameters of model PW-DU is by defining a new parameter *b* ∈ [0, 1] as the distance of *β* ∈ [0, 2] to the optimal value of 1 (smaller *b*—more optimal). The results of fitting the regression model *time*_*i*_ = *α*_0_ + *α*_1_*τ*_*i*_ + *α*_2_*γ*_*i*_ + *α*_3_*b*_*i*_ + *e*, are shown in Table 4, showing slope coefficients for *γ* to be significant in line with our hypothesis.

**Table 4:** Linear regressions predicting decision times by parameters fitted by model PW-DU.

Times	*p*	F-statistic	*α*_0_	*α*_1_ (*τ*)	*α*_2_ (*γ*)	*α*_3_ (*b*)
Initial	.001	F(3,2320)=5.23	2587	-57	337	467,
				*p* = .5	*p* = .005	*p* = .001
Subsequent	< .0001	F(3,6043)=11.15	985	32	-92	111
				*p* = .07	*p* = .0006	*p* = .0006

In summary, these results show that (1) discounting is positively correlated with Initial decision times, meaning that longer deliberation results in a longer planning horizon, (2) discounting is negatively correlated with Subsequent decision times, meaning that a longer initial deliberation results in fewer decisions made online and (3) discounting explains decision times beyond measuring noise in utility computation.

## Discussion

We examined how people plan under uncertainty in a spatial setting using a novel Maze Search Task (MST), which requires people to navigate partially observable mazes in search of a randomly placed exit. We designed and evaluated a family of computational models that plan ahead, along with a family of myopic heuristics that choose the next observation one step at a time, inspired by studies of information search. In addition to limited planning horizon, which has been explored in previous work although not in spatial planning tasks, our planning models incorporate number and probability perception, which were not previously evaluated in the context of planning.

We found that people’s decisions in the MST were best explained by models that plan, as opposed to myopic heuristics, but human plans tend to deviate from the normative Expected Utility model. While our results do not allow us to declare an overall clear ‘winner’ model, we can identify necessary properties that a planning model must have to explain human behavior. Specifically, we find strong evidence in favor of a limited planing horizon, in three parts: (1) the models with the best fit to human behavior all include a discount rate parameter, (2) people’s response times predict their fitted discount rate, and (3) in special cases where probabilities and costs are kept equal, only discounting can explain human preferences. This finding is consistent with previous studies of non-spatial tasks [1, 2, 14]. At the same time, our results do not exclude the influence of number or probability perception, as models parameterized with Probability Weighting and Numerosity perception show a good independent fit to people, suggesting that more future work is needed to understand how exactly various cognitive and perceptual factors interact in spatial planning.

Beyond demonstrating limited horizon in spatial planning, we do not argue that one of the planning models should be a single winner at predicting how people plan, as individual differences in the MST suggest that multiple planning strategies may be available to people at the same time (this point is similar to previously reported differences in decision-making [22, 42]). Our results demonstrate that planning strategies vary significantly across individuals, and illustrate the strengths of our computational modeling approach that evaluate multiple models, as a method of understanding diversity of human cognitive behaviors in realistic tasks. This result adds to a growing trend in cognitive science literature where large families of models are used to explore the distribution of cognitive strategies across individuals [29, 30]. In this regard, increasing expressiveness of the hypotheses class if of a practical value for developing theories in the cognitive domain. In future work we intend to further explore the possibility that multiple planning strategies may be available to individuals, and that individuals may switch between these strategies, as well as learn new strategies online, as they adapt their cognitive resources to the changing demands of a task.

While we examine planning in decision-trees with maximal depth of 10 (corresponding to mazes with up to 10 rooms and an order of 2^1^0 states), future studies could investigate how discounting interacts with perceptual approximations in larger problems as the computational complexity of the problem increases. In particular, several studies show that practice can increase planning horizon in games such as Chess and Four-in-a-row, in terms of the number of nodes explored by a model [16, 17], which suggests that humans are so skilled at realistic tasks, such as navigation, at least partly due to extensive practice. This rises the question of whether practice would similarly improve performance in MST, in terms of planning depth measured as discount rate, and to what extent such an improvement could transfer to other tasks.

Additionally, studies have shown that performance in high-level cognitive tasks, such as tasks that require learning, is relatively immune to financial incentive, in contrast to tasks that require a fixed strategy [43]. This result rises the question of how motivation might impact performance in Maze Search, suggesting that increasing motivation could lead to faster reaction times in people who use heuristics, but is unlikely to impact the depth to which people plan. Further, affective states, such as mood and arousal, are known to influence performance across a variety tasks [44], although the effects of affect of planning are not yet addressed. A computational account of how affective states impact planning in MST could guide design of mental state inference algorithms, and advance our understanding of cognitive deficits in people with mental health disorders.

The planners built in this work do not purport to describe all there is to spatial planning, or to exhaust all possible ways in which people could make choices in MST. Our modeling goal in this work was to contrast a set of naturalistic planning models motivated by prior literature against myopic heuristics motivated by literature as well as by participant feedback received during pilots. While it is possible to imagine more sophisticated heuristics, for example based on symmetries [45] or hierarchical representations [46, 47], such heuristics would serve to reduce complexity of the task rather than support myopic decision-making.

Further, while our results suggest that probability weighting may play a role in spatial planning, we note an overlap in the scope of behaviors that can be modeled by discounting and by probability weighting in our experiments, motivating further study of cognitive mechanisms of probability perception in spatial domains. One direction of such a study is to examine whether people mentally represent probabilities in the same way across spatial and non-spatial contexts, such as, investigating whether probability weighting generalizes between MST and monetary gambles studied by Prospect Theory [31]. Another direct prediction of Prospect Theory that can be studied in MST is the phenomenon of *subadditivity*—an observation that weighted probabilities of different outcomes of an event do not add up to 1 [48]. The extent of subadditivity depends on the parameter *β*, which we fit to participants, where *β* → 0 produces greater underestimate in cumulative probabilities of disjoint events. For example, consider an agent choosing between two maze areas with objective probabilities of {0.1, 0.9}. Given *β* = 0.7 (the mean value of *β* fitted to people in Experiment 1 with two-room mazes) the sum of weighted probabilities is 0.98. In this case, only a minimal subadditivity is implied by the model. For *β* = 0.35 (the mean value of *β* fitted to people in Experiment 2 with big mazes) the sum of weighted probabilities is 0.9, which implies a more substantial subadditivity.

One possible explanation for observing a greater subadditivity in larger compared to smaller mazes, is that subadditivity could reflect the assumption that there likely exist potential outcomes unknown to the agent. As this likelihood should increase with the increasing cost of forming a mental world-model in bigger mazes, people may be more inclined to down-weight the estimated probabilities of the known outcomes to leave room for this uncertainty. Preliminary empirical support for this hypothesis comes from the previously observed relationship between the extent of individual subadditivity and working memory capacity [49], but further work is needed to test whether subadditivity in realistic scenarios may be arising from the mental cost of forming a mental map representation.

While we used analytical models for methodological rigor, future studies can investigate algorithmic models that sample a small number of paths, which could be particularly well suited for large environments. Future studies can also investigate the stability and generalization of individual planning strategies between tasks, and over time. Do people learn to plan by learning a library of task-specific strategies (e.g. Chess strategies [17]), or by learning abstract cognitive mechanisms for planning [15, 50], for example by learning to plan further ahead?

Lastly, we note several limitations of our modeling approach. In our analysis, all decisions are treated as Markovian, meaning that each decision is independent of whether a certain maze state is encountered as a new maze (as root of a decision tree), or as a part of a partially explored maze (one of the sub-trees). While this assumption is common in computational models of planning, future studies could test whether in practice individuals make consistent choices after partially exploring a maze, compared to when an identical partially revealed maze is presented as a new trial. This consistency (or the lack of it) could indicate to what extent people plan a search policy, or engage in online updating.

## Conclusion

Our work takes a step toward computationally understanding and modeling an important, real-life domain of planning: how humans plan in spatial multi-step contexts. The results discussed in this work have implications for the study of human cognition, as well as for building an AI that can interpret human planning to infer goals, offer assistance, and support human-AI collaboration. Our work makes a novel theoretical contribution, by demonstrating evidence of limited planning horizon in spatial planning tasks, and exploring the possibility that in human planning action values may be also affected by cognitive mechanisms of numerosity and probability perception. It also makes a methodological contribution, illustrating the value of using a multi-model approach for understanding cognition. and illustrating the value of our multi-model approach for understanding cognition. Beyond our theoretical and empirical contributions, we hope our MST methodology can become a tool for exploring a larger variety of planning environments, cognitive models and algorithms.

## Supporting information

S1 Appendix Planning state-space. (PDF)

S2 Appendix Planning models. (PDF)

S3 Appendix Examples of value functions behaving differently. (PDF)

S4 Appendix Experiment 1, additional results. (PDF)

S5 Appendix Experiment 2, additional results. (PDF)

S6 Appendix Maze search task. (PDF)
