# Supplementary material for: Approximate planning in spatial search
Source: PLoS Comput Biol. 2024 Nov 12;20(11):e1012582. doi: 10.1371/journal.pcbi.1012582 (PMC11584085; doi:10.1371/journal.pcbi.1012582)
Supplement: S1 Appendix — (PDF) [file pcbi.1012582.s001.pdf]

S1 Planning state-space

We based our computational approach on choosing a path through a decision tree that maps states to specific observations within a maze. This design is supported by an empirical evidence that people take direct routes between observations (see Figure S1), even through the grid-world layout of MST in principle allows players to take indirect routes, or even indefinitely move between any adjacent empty tiles without making any observations. People's tendency to take direct routes between observations strongly suggests that people use efficient problem representations, in line with previous work [47].Our decision tree model is further supported by the empirical distribution of human decision times in different types of tiles inside a maze, as shown in Figure S2. The figure shows that people move quickly when traveling between observations (in Corridors) and take longer to make a move whenever new hidden tiles are revealed (Decision). The longest decision time occurs at the initial starting location (Start), where people may study the map and plan their path before moving. Motor errors, that is, overshooting the end of a step sequence that connects observations by a step, and immediately going back, occur on about 1% of corridor moves (see Figure S1).

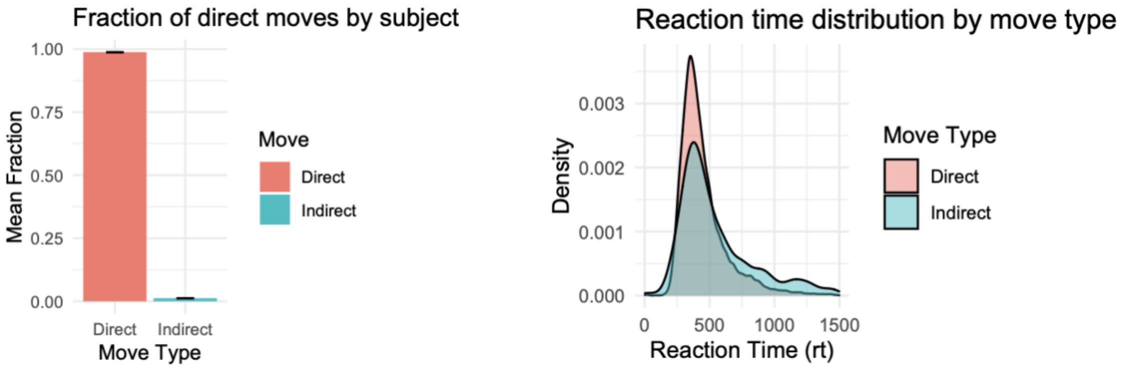

Figure S1: Distribution of decision times in corridors while moving toward the next observation by an optimal route (Direct) and while deviating from the optimal path (Indirect). Of the corridor steps, 99% lie on optimal routes to room-revealing states, and the remaining 1% are motor errors.

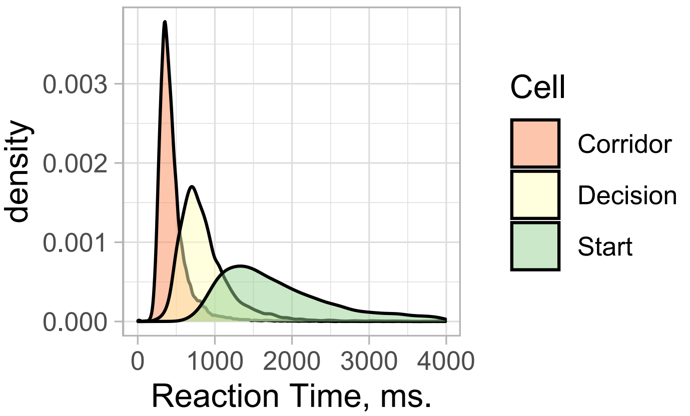

Figure S2: Distribution of decision times in Experiment 1, measured in milliseconds, as humans click on maze tiles, plotted by location type: Start - initial decision time to make the first move at the start of the trial, during which people form a mental representation of a maze and plan a search within a certain planning horizon (or choose where to observe next using a step-wise heuristic); Corridor - moving between observations; Decision - subsequent decision time, corresponding to inner nodes of the decision tree, where people either make a pre-planned move, or decide where to observe next using a step-wise heuristic. For clarity, only times under 4000ms are shown, as the longest decision times in distributions' tails take minutes.
