# Supplementary material for: Approximate planning in spatial search
Source: PLoS Comput Biol. 2024 Nov 12;20(11):e1012582. doi: 10.1371/journal.pcbi.1012582 (PMC11584085; doi:10.1371/journal.pcbi.1012582)
Supplement: S2 Appendix — (PDF) [file pcbi.1012582.s002.pdf]

## S2 Planning Models

### S2.1 Probability Weighted Utility

We used probability weighting of the form $p = exp(-1(-\log(p))^{\beta})$, the shape this function takes for different $\beta$ is shown in Fig.S3. The original Prospect Theory sets $\beta \in [0, 1]$. Here we also consider $\beta \in [0, 2]$ where values of $\beta \in (1, 2]$ imply overweighting large probabilities.

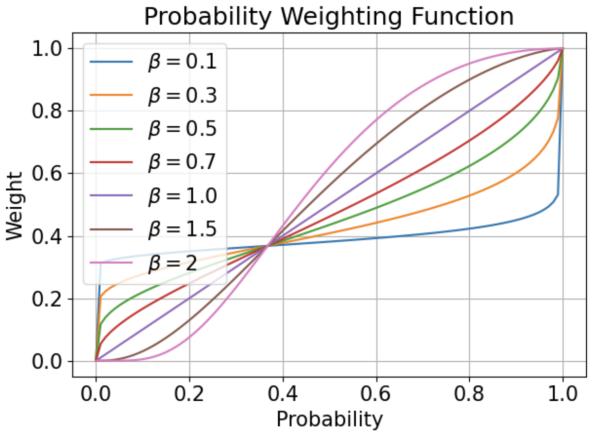


Figure S3: The probability weighting function for different $\beta$.

Probability Weighted with $\beta < 1$ makes room of different size appear to be more alike, and with $\beta > 1$ makes room of different size appear to be more different. For example, suppose that a maze with 10n unobserved cells includes rooms of sizes n and 3n, and suppose that a person searches this maze exhaustively, so that the first decision (there are still a number unobserved rooms in the maze), and the last decision (only two unobserved rooms remain) both requires choosing between rooms of size n and 3n. In the first decision, the probabilities associated with the rooms are (0.1, 0.3). In the last decision they are (0.25, 0.75). Then, different values of $\beta$ will transform these probabilities as shown in Figure S4

### S2.2 Numerosity perception in Maze Search Task

The information-theoretic numerosity model assumes that the systematic deviation in the perceived number is due to processing limited bits of information, where the prior distribution over observed numbers favors small quantities. FigureS5 (left) shows how the number of tiles perceived by people is predicted to change with the number of bits of information processed.

A indirect effect of numerosity model, is a distortion of probability perception. This happens because probabilities are computed as ratios of revealed to remaining tiles. For example, Figure

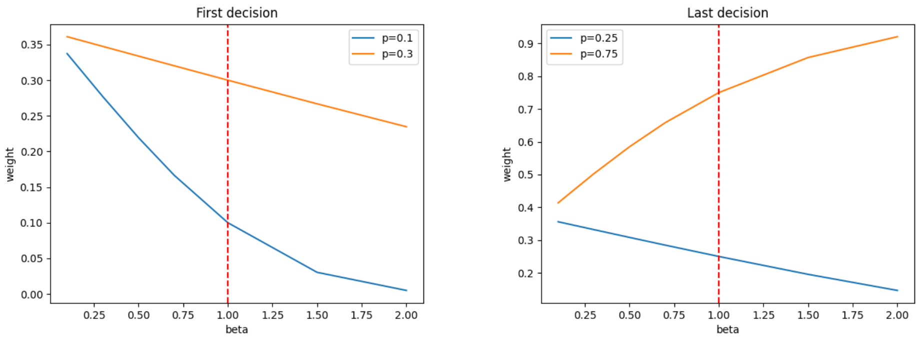


Figure S4: Perceived probability for rooms of different sizes, at the beginning and at the end of a trail, where the room size ratio is 1:3. The red line at $\beta = 1$ indicates no probability weighting.

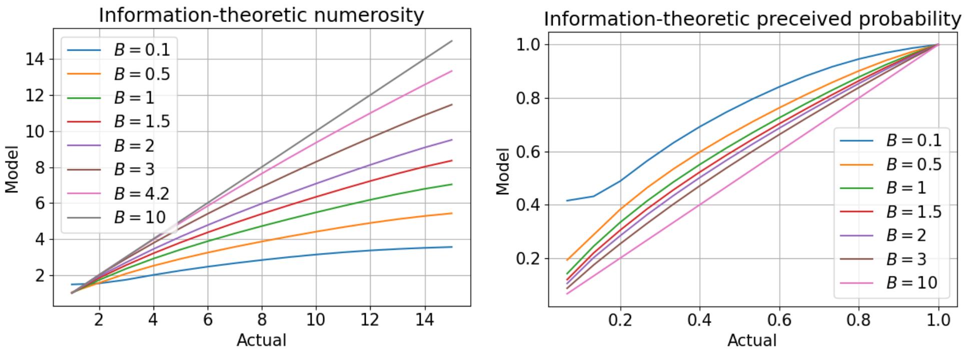


Figure S5: Left: Number of tiles perceived under the model plotted against the actual number. Right: Perceived probability predicted by information-theoretic numerosity model plotted against the actual probability, assuming the exit is equally likely to be in any hidden tile.

S5 (right) shows subjective probabilities estimated using different $B$, assuming a total of 15 unobserved tiles, and various sizes of rooms. The effect of this distortion is different from the effect of the PW model, as modeling Numerosity slightly inflates the small and middle probabilities, while leaving large probabilities unchanged.

## S2.3 Monte-Carlo Tree Search - Sampling Model

**Algorithm 1:** Monte Carlo Tree Search for MST

**Data:** Tree to determine best child of node $N$

Initialize: Decision node $N$, $Budget$
$k \leftarrow 1$ **while** k $\leq$ Budget **do**
    $n \leftarrow N$
    **while** node n is not a leaf node **do**
        $n \leftarrow max_{c \in C(n)} ubc(c)$
    **end**
    **if** node $n$ has been visited **then**
        add children nodes of node $n$
        $c \leftarrow$ random child of $c$
    **end**
    **while** p $\sim$ Uniform(0,1) $>$ P(exit found at c) **do**
        $c \leftarrow$ random child of $c$
    **end**
    value of node $n \leftarrow$ value of node $n + c$
    number of visits to node $n \leftarrow 1 +$ number of visits to node $n$
    $k \leftarrow k + 1$
**end**

The Sampling model approximates the EU model with increasing accuracy as the budget parameter is increased. Figure S6 shows the probabilities of actions predicted by the EU and Sampling model, correlation $r = 0.95$, assuming budget and exploration parameters are chosen to maximize the correlation, and EU is parameterized with $\tau = 1$. The optimal exploration parameter depends on the budget, with larger budgets typically requiring higher exploration parameter to achieve best approximation.

## S2.4 Defining Maze Search using the POMDP formalism

While we do not solve Maze Search by Reinforcement Learning, it is possible to define the problem using a Partially Observable Markov Decision Process (POMDP) formalism, as we show below. This formulation is closely related to formulation of [1] and [2].

The agent occupies a discrete state space $X$ of cells in a 2D grid, where each cell can contain a visible wall, visible floor, unobserved floor (which could hide the Exit), or visible exit. The hidden environment state $Y$ is the set of possible assignments of exit to the unobserved floor cells. Possible actions include North, South, East, and West. Valid actions yield the intended transition with probability 1 and do nothing otherwise; invalid actions (e.g., moving into walls)

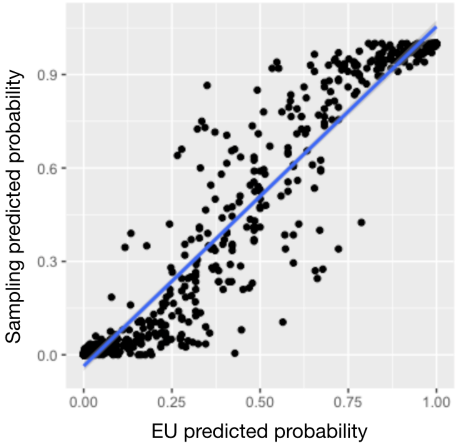


Figure S6: Correlation between action probabilities predicted by EU and Sampling.

have no effect on the state.

The agent has a 360 degree vision, and can see any cells not occluded by walls. In other words, agent's visual observations are represented by the discrete grid isovist from the agent's location, as described in the main text. The observation distribution $P(o|x, y)$ encodes which environments in $Y$ are consistent with the contents of the isovist from location $x$. Bayesian belief updating at time $t$ is a deterministic function of the prior belief $b_{t-1}$, the observation $o_t$ , and the world state $< x_t, \mathbf{y} >$. The agent's updated degree of belief in environment $y$ satisfies $b_t(y) \propto P(o_t|x_t, \mathbf{y})b_{t-1}(\mathbf{y})$.

The agent's reward function $R(x, y, a)$ encodes the subjective utility the agent derives from taking action $a$ from the state $< x_t, \mathbf{y} >$, where rewards result from reaching the exit and are the same in each maze, and the costs are incurred by taking actions (e.g. for the optimal agent, an action that moves one step incurs a cost of 1). The agent's POMDP is defined by the state space, the action space, the world dynamics, the observation model, and the reward function. The agent's policy is stochastic, given by the softmax of the lookahead state-action value function $Q_{LA}$: $P(a|b, x, y) \propto exp(\tau Q_{LA}(b, x, y, a))$. The $\tau$ parameter establishes the degree of determinism with which the agent executes its policy, capturing the intuition that agents tend to but do not always follow the optimal policy. Here $_LA$ can be represented by different cost models that may include additional parameters. The value function computes the expected utility of an action in terms of how many steps away the agent is from the expected location of the exit as a result of taking this action:

$Q(a, < x_t, \mathbf{y} >) = \sum_X P(Y)_t R(x, y, a) + \gamma \max_{a_i \in A}\{Q(a_i, < x_{t+1}, \mathbf{y} >)\}$

where $\gamma$ is a discount factor, similar to discounting used in our original formulation, but

conceptually different as it will scale the paths between observations proportionally to their length. Finally, the reward function can be defined to depend on the size of the observed areas and distances between them, assuming a step cost function $step(x, y, a)$ and a reward for observing a given area given by a function $cells(x, y, a)$

$R_{EU}(x, y, a) = cells(x, y, a) - step(x, y, a)$

# References


[1] Chris Baker, Rebecca Saxe, and Joshua Tenenbaum. Bayesian theory of mind: Modeling joint belief-desire attribution. In Proceedings of the annual meeting of the cognitive science society, volume 33, 2011.

[2] Marta Kryven, Tomer D Ullman, William Cowan, and Josh Tenenbaum. Outcome or strategy? a bayesian model of intelligence attribution. In CogSci, 2016.
