# Supplementary material for: Approximate planning in spatial search
Source: PLoS Comput Biol. 2024 Nov 12;20(11):e1012582. doi: 10.1371/journal.pcbi.1012582 (PMC11584085; doi:10.1371/journal.pcbi.1012582)
Supplement: S3 Appendix — (PDF) [file pcbi.1012582.s003.pdf]

S3 Examples of value functions behaving differently

Figures S7-S14 show mazes where different models take different paths through a maze. Figures S7, S8 show a simple two-choice example, in which all models and heuristics, except for DU, are indifferent between moving toward "1" or "2" from the starting location (marked "S"). Figure S8 shows in detail the probabilities of choosing "1" and "2" by each model, deepening on model parameters. Each subplot in figure S8 shows a different model, showing that models without discounting assign probability 0.5 to choosing each direction.

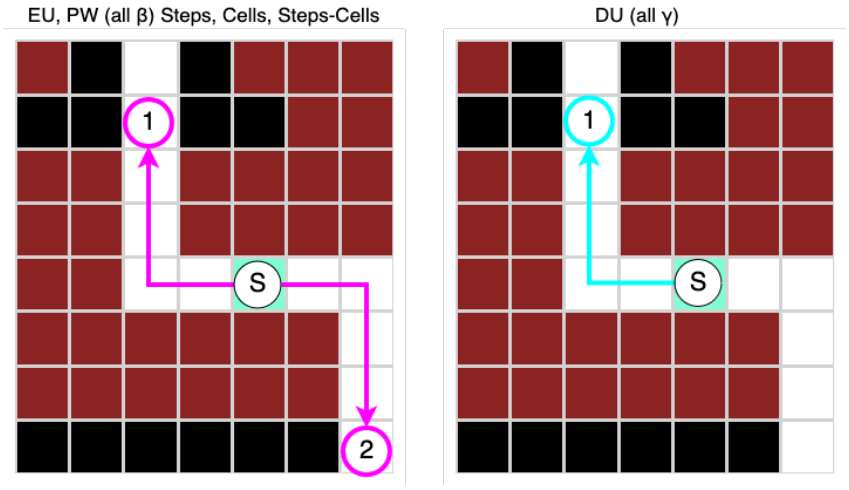

Figure S7: A maze from Experiment 1, with paths predicted by different models. The DU model prefers searching room ‘1’ first, while the other models are indifferent between ‘1’ and ‘2’.

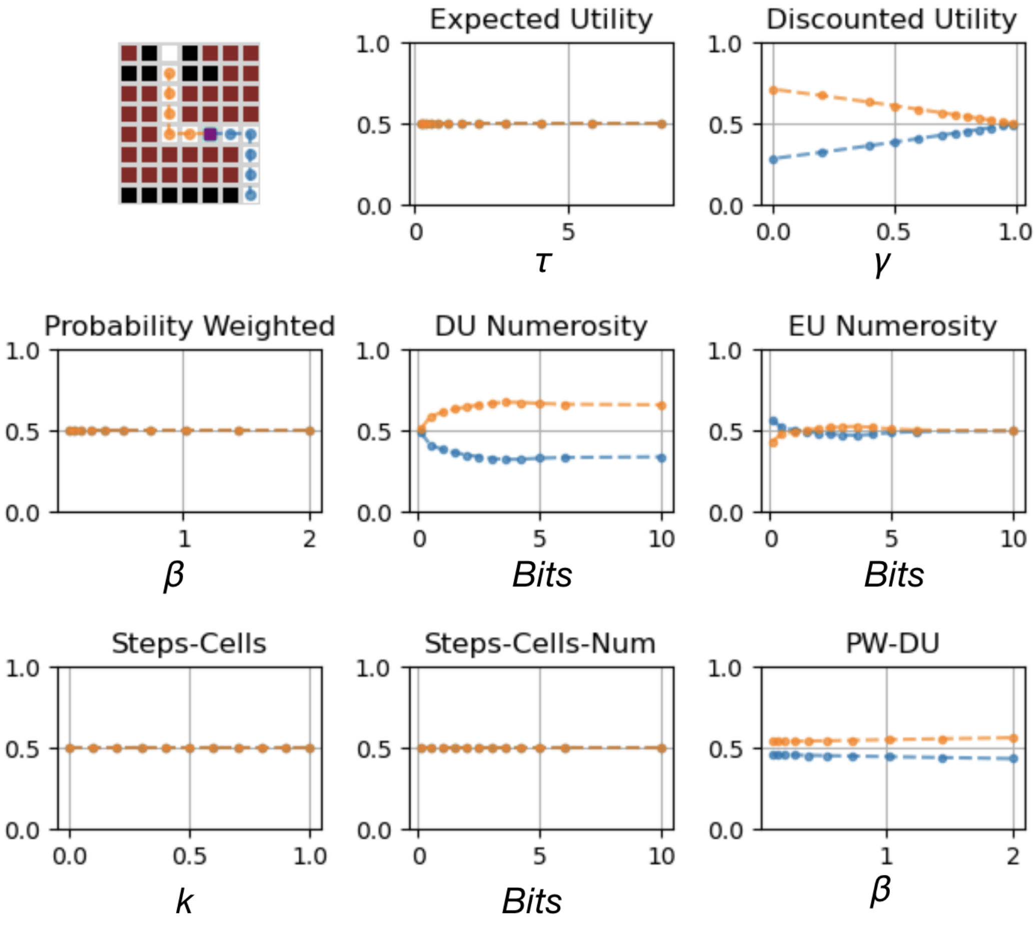

Figure S8: Models predicting different paths in maze from Figure S7. The starting location is marked by a purple square. Only models parameterized with discounting can capture human preference for going up. The X axes show model parameters. The y-axis shows probabilities assigned by different models to path directions. For models with several parameters, the values of parameters not shown are fixed to the participant mean in the experiment.

Figures S9, S10 and S11, show a maze in which distinctly different behaviors are predicted by EU, PW ($\beta = 2$), PW ($\beta = 0.1$) and DU. Figure S9 shows the most likely path, according to each model, and Figure S10 shows in detail the probabilities of each possible choice by each model in the initial decision state (the starting location is marked by a purple square), depending on model parameters. Figure S11 shows predicted choice probabilities in one of the subsequent decisions within the same maze. In this example, the difference between the DU and the PW ($\beta = 2$) models comes from a downstream decision where the EU model prefers to go Up as it prioritizes a shorter path to the next observation, wile the PW model is indifferent between Up and Left directions, as illustrated in Figure S11.

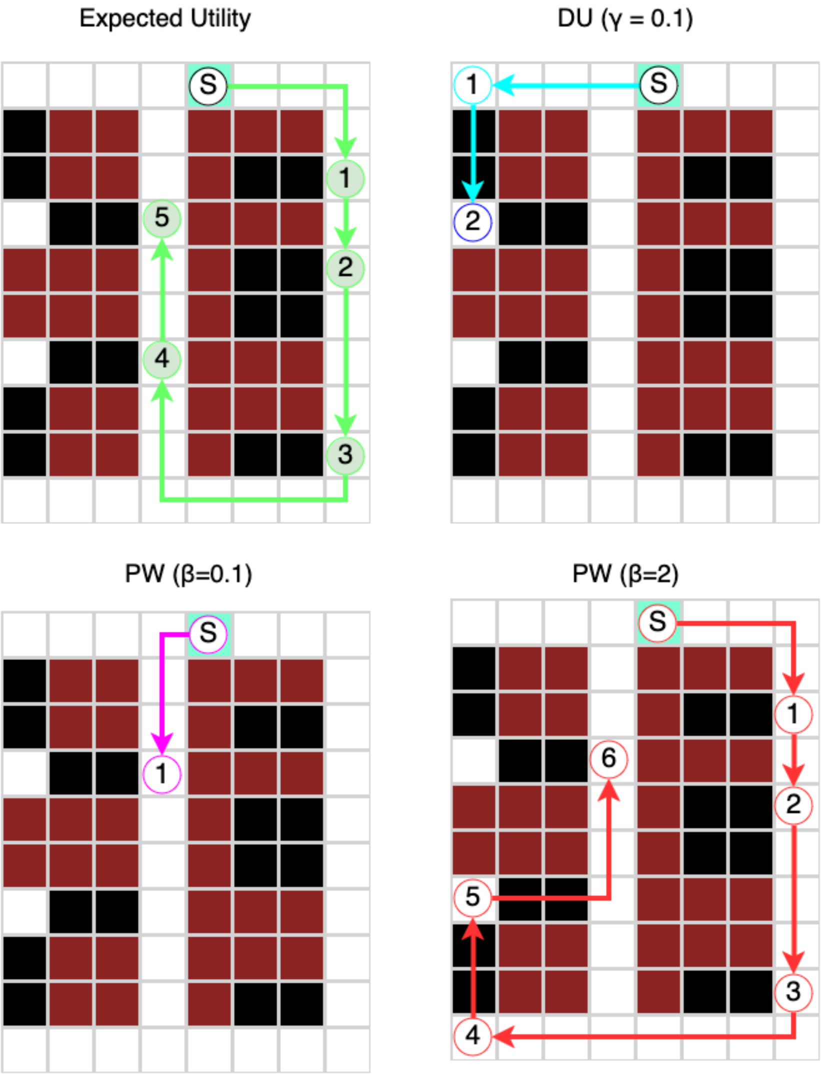

Figure S9: An example of a maze from Experiment 2, in which different models predict different path through a maze. The EU model makes the initial decision to move toward the bigger room, taking 5 steps. Model PW with a small β sees rooms of different size as similar, meaning that this model goes to one of the closer rooms, taking 4 steps. The difference between the DU and the PW models comes from a boundary case, where the DU model becomes indifferent between two equidistant rooms when $\gamma - > 0$.

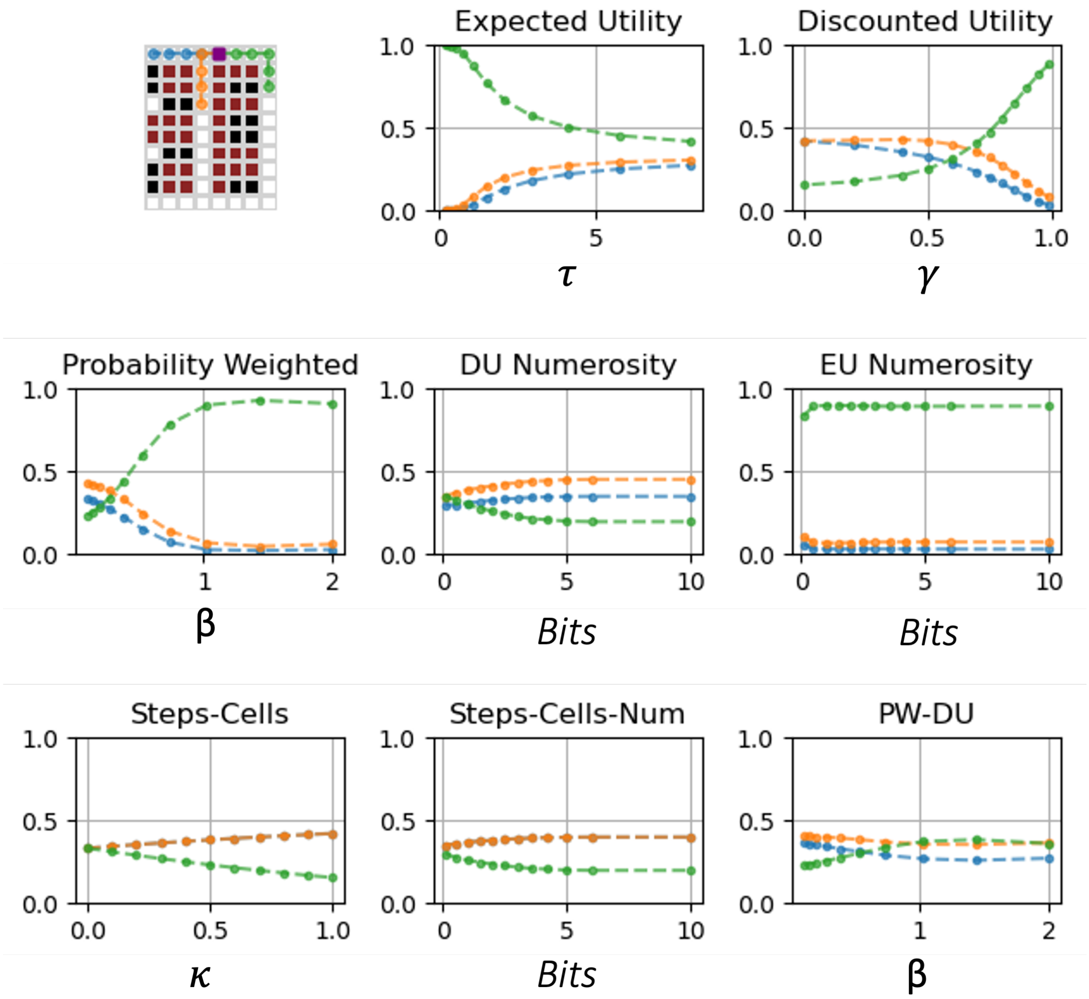

Figure S10: Likelihood of different directions being chosen by the different models based on model parameters. Each graph shows the likelihood of choosing each direction by a different model. The X axes show model parameters. The y-axis shows probabilities assigned by models to the actions. For models with several parameters, the values of parameters not shown are fixed to the participant mean in the experiment.

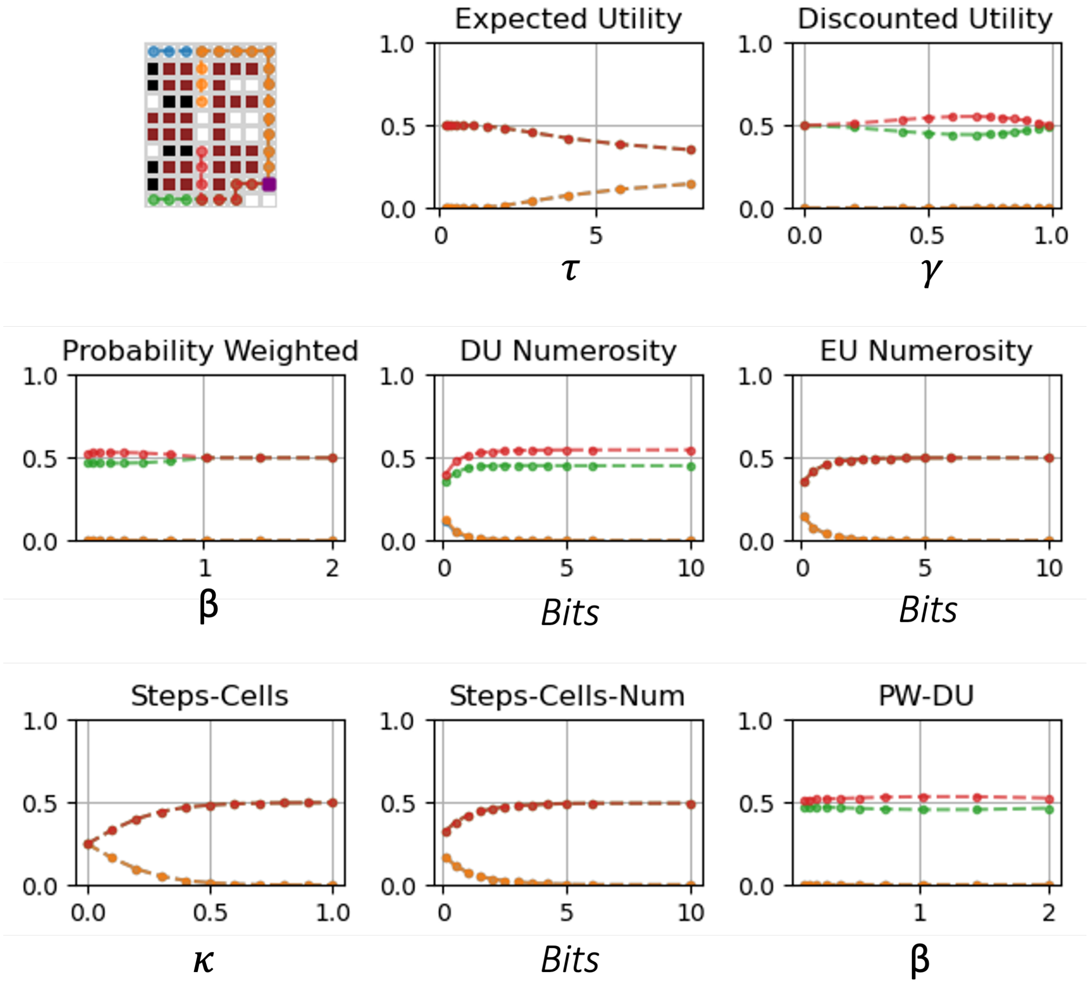

Figure S11: Likelihood of different directions being chosen by the different models based on model parameters. Each graph shows the likelihood of choosing each direction by a different model. The X axes show model parameters. The y-axis shows probabilities assigned by models to the actions. For models with several parameters, the values of parameters not shown are fixed to the participant mean in the experiment.

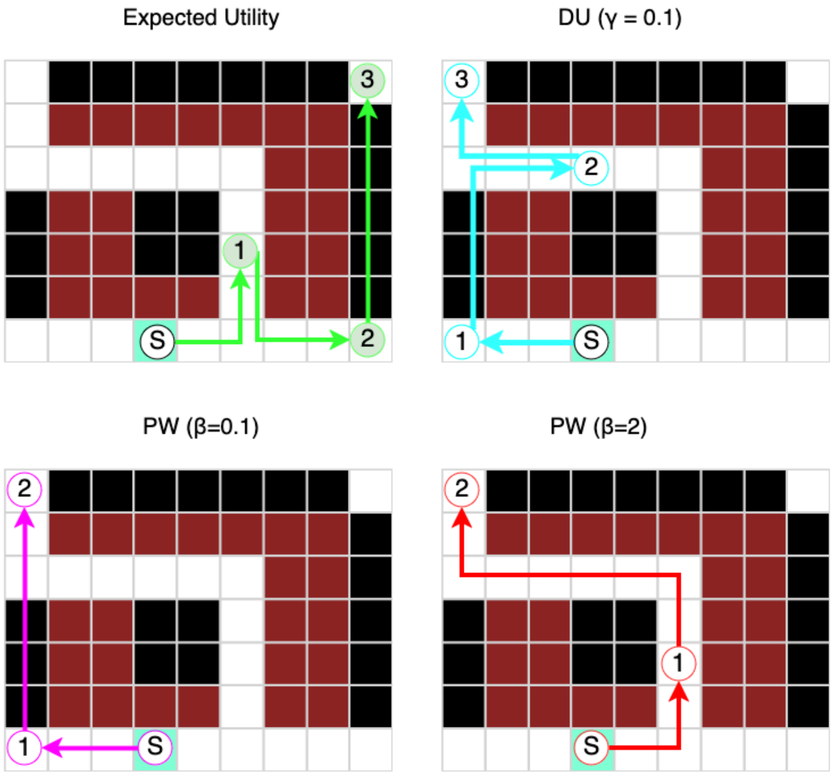

Figure S12: Different models predict different path through a maze.

Figures S12, S13 and S14 show another maze where four models take different routes. The most likely paths according to each model are shown in Figure S12, and probabilities assigned to actions by different models during specific decision shown in Figures S13 and S14.

Figure S14 illustrates an interesting property of the DU model, where given a small γ the model prefers *a longer path toward a smaller room*. This happens because the four cells that the DU chooses to open are relatively closer to the agent – if the exit is found within those four cells, then it will be on average 2 steps away. In contrast, if the exit is found within the seven cells on top of the maze (this choice is preferred by all other models), it will be on average 4 cells away. So, the DU model can behave similarly to the Steps heuristic, while also being sensitive to the shape of the revealed rooms.

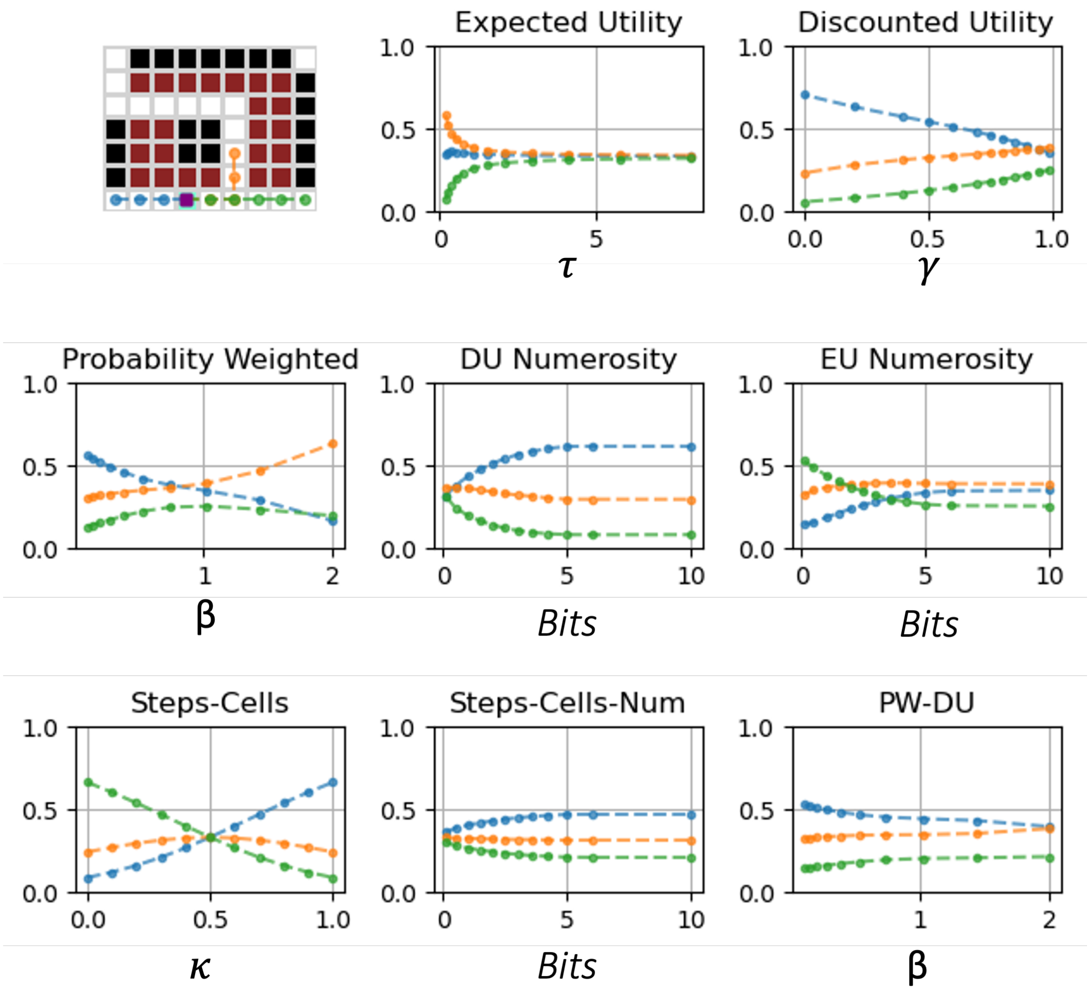

Figure S13: Likelihood of different directions being chosen by the different models, based on model parameters. This figure shows the initial decision, seen by all models. The X axes show model parameters. The y-axis shows probabilities assigned by different models to path directions. For models with several parameters, the values of parameters not shown are fixed to the participant mean in the experiment.

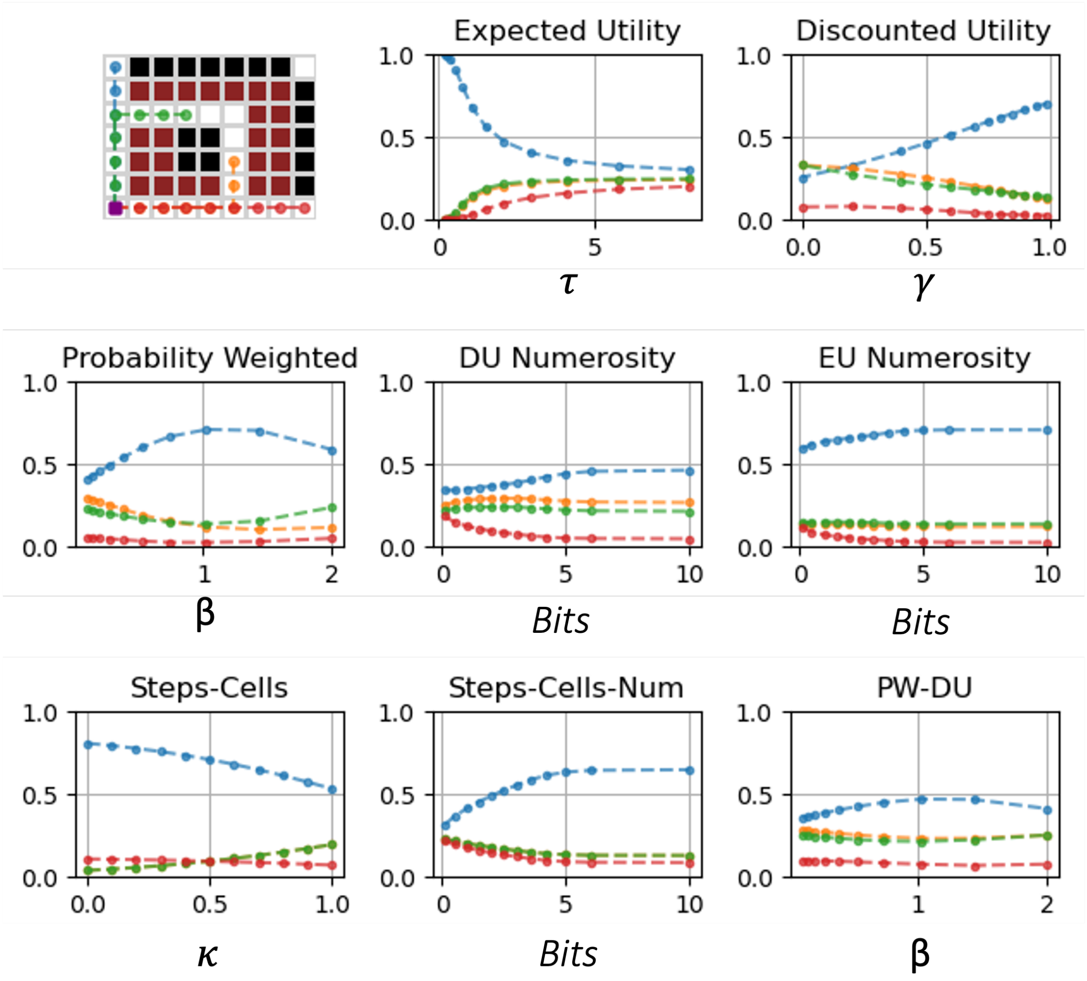

Figure S14: Likelihood of different directions being chosen by the different models, based on model parameters. This figure shows a second a decision in the path. Only models PW ($\beta = 0.1$) and DU ($\gamma = 0.1$) reach this decision. The X axes show model parameters. The y-axis shows probabilities assigned by different models to path directions. For models with several parameters, the values of parameters not shown are fixed to the participant mean in the experiment.
