# Supplementary material for: Approximate planning in spatial search
Source: PLoS Comput Biol. 2024 Nov 12;20(11):e1012582. doi: 10.1371/journal.pcbi.1012582 (PMC11584085; doi:10.1371/journal.pcbi.1012582)
Supplement: S4 Appendix — (PDF) [file pcbi.1012582.s004.pdf]

S4 Experiment 1, Additional results

S4.1 Binary choice mazes

Experiment 1 includes 23 mazes with strictly binary trees (mazes with two observations, and no loops). These mazes were designed to contrast room distance and room size, such that bigger rooms require more steps to reach them. In terms of Expected Utility of these choices, the mazes are designed so that (1) expected value can not be predicted from distance or size differences between rooms (shown in Figure S15) (2) The Expected Utility of the bigger and further room is larger or equal to that of the closer and smaller room. (e.g. Figures S17, S18, S19) The second point is important, as people tend to prefer closer rooms more often than the EU model (Figure S16).

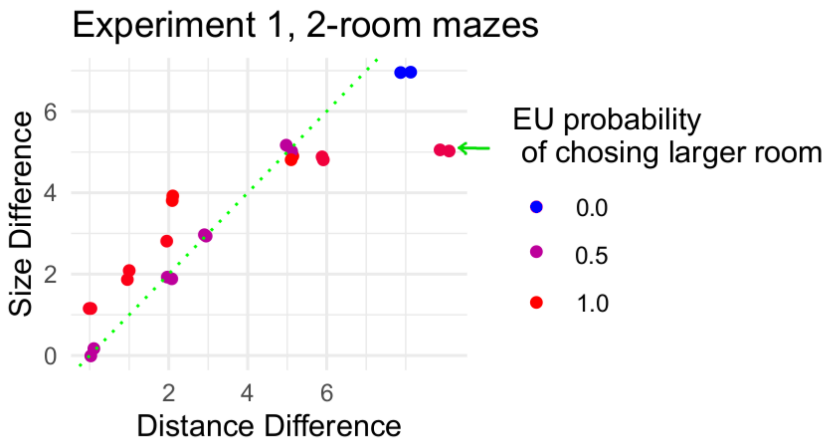

Figure S15: Expected Utility of binary choices in Experiment1. The figure is generated with a minimal decision temperature, to illustrate the preferences of the EU model. The points are jittered for readability. The arrow points to a maze with the largest difference in distance between rooms, additionally shown in Figure S17

To illustrate how step and size differences are not sufficient to explain expected value, consider the two examples below. In both examples, the differences between size and distance are 5 cells and 5 steps, but EU prefers a larger room in one case, and is indifferent between the two directions in the other case in the other case.

In the figure below, EU prefers to search the room with 6 cells, vs the room with 1 cell (difference of 5 cells). The distances to these rooms are 4 and 9 steps (difference of 5 steps). Here, 0.58 of participants explore the larger room, in agreement with EU:

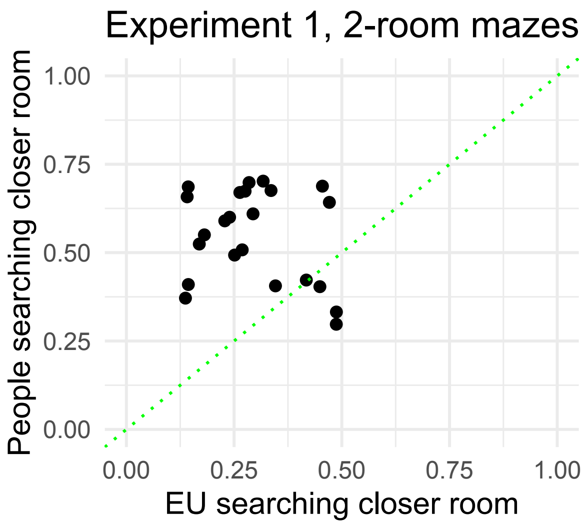

Figure S16: EU and human preferences for the 23 binary mazes from Experiment 1. Compared to EU, people are more likely to search the closer room first.

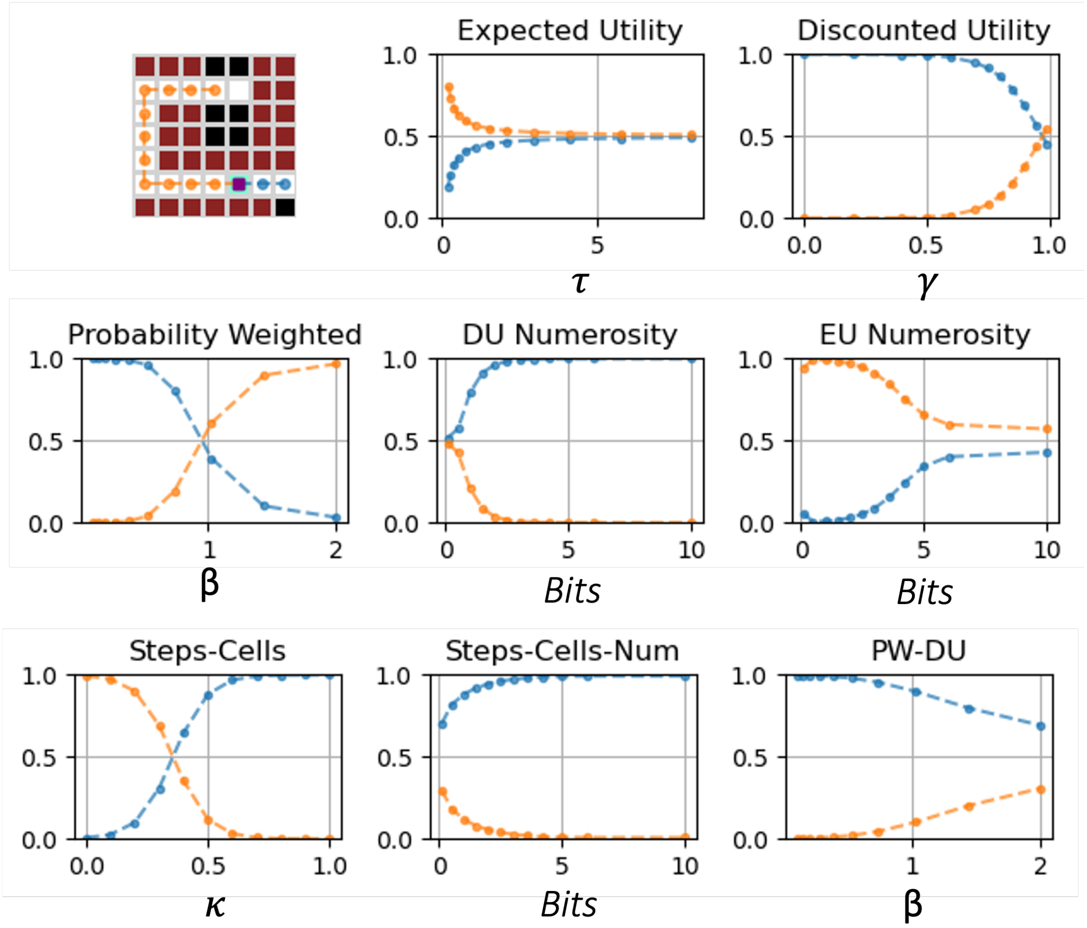

Figure S17: A maze from Experiment 1 where the EU model prefers to search the larger room first, but 0.67 of participants preferred to search the smaller room first. Different plots show probabilities of choosing either path by different models. The X axes show model parameters. The y-axis shows probabilities assigned by different models to path directions. For models with several parameters, the values of parameters not shown are fixed to the participant mean in the experiment.

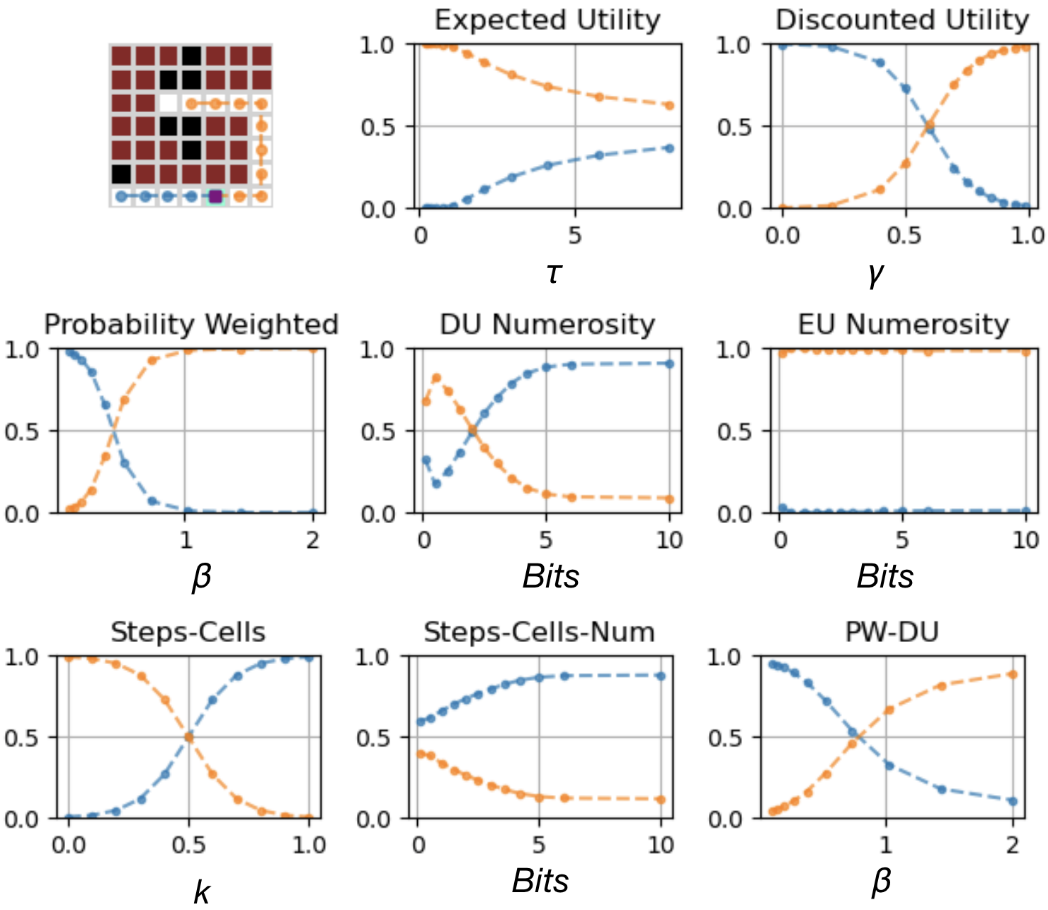

Figure S18: A maze from Experiment 1 where the EU model prefers to search the larger room first, in agreement with 0.58 of participants. Different plots show probabilities of choosing either path by different models. The X axes show model parameters. The y-axis shows probabilities assigned by different models to path directions. For models with several parameters, the values of parameters not shown are fixed to the participant mean in the experiment.

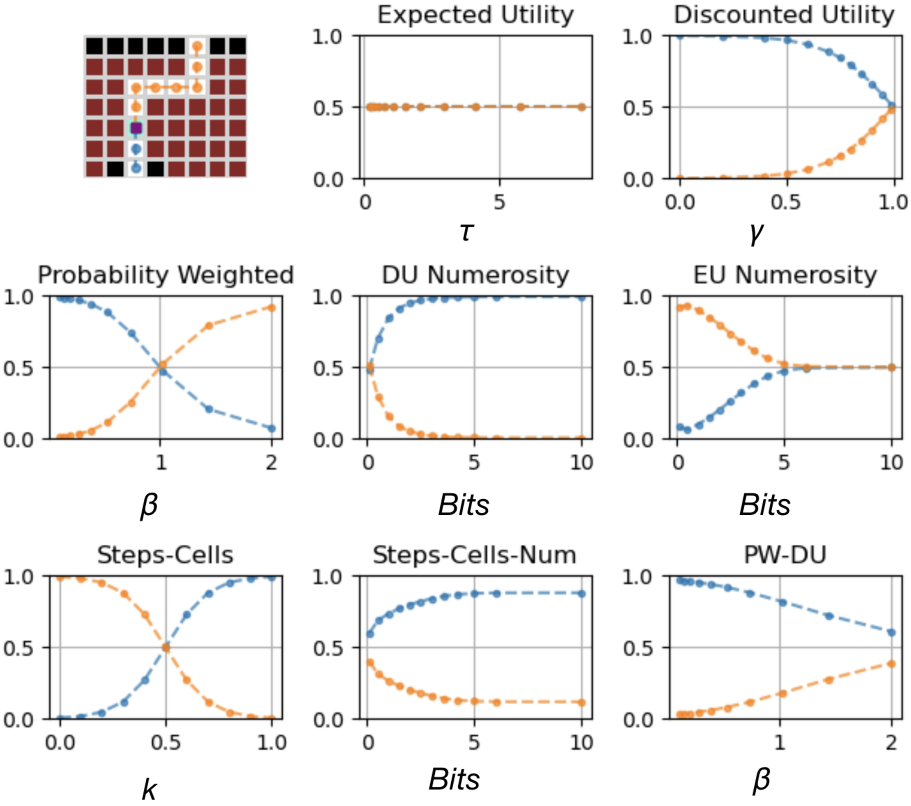

Figure S19: A maze from Experiment 1 where the expected utility of both directions is the same, but 0.6 of participants have a preference for searching the smaller room first. Different plots show probabilities of choosing either path by different models. The X axes show model parameters. The y-axis shows probabilities assigned by different models to path directions. For models with several parameters, the values of parameters not shown are fixed to the participant mean in the experiment.

In Fig.S19, expected utility of both directions is the same. The rooms contain 2 and 7 cells respectively (difference of 5 cells). The distances to these rooms are 2 and 2 steps (difference of 5 steps). Here, only 0.4 of participants explore the larger room.

S4.2 Fitting models with Monte-Carlo Cross validation

Monte-Carlo cross-validation is a method of fitting models over multiple bootstrapped iterations (we run 100) which allows us to obtain confidence intervals on each individual's LLs. Figure S20 A. shows the mean LL per decision as model fit, with 95% CI over participants. Figure S20 B. illustrates variability between individuals. For each indivudual we first determine the best fitting planner and the best-fitting heuristic, and plot the LL of best heuristic and best planning model for each individual with 95% CI. The labels "Heuristic", "Planning", and "Not defined" are assigned based on whether the 95% CI of the LL for best-fitting planner and heuristic overlap. Note that this method may overestimate the number of "Not defined" individuals, as we bootstrap the 95% CI of the mean, not the 95% CI of the *difference* between means. Figure S20 C. summarizes the number of individuals presented in each category in Figure S20 B. in a bar plot. The distribution of best fitting models across individuals is shown in Figure S23.

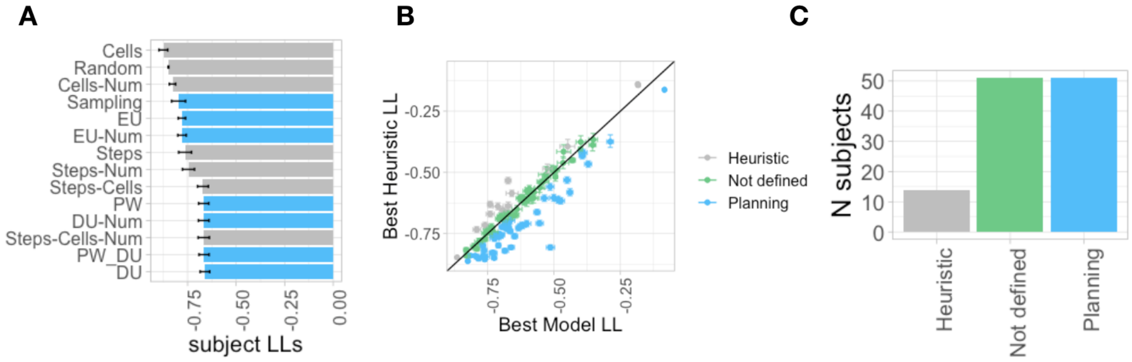

Figure S20: Experiment 1. Models fitted with Monte-Carlo cross-validation. **A.** Mean LL per decision with 95% CI, averaged over participants. **B.** Mean LL per decision for an individual's best-fitting planning model and best-fitting heuristic. Each dot represents an individual, error bars are 95%CI. Participants for whom CIs of heuristic and planning model do not overlap are labeled as "Heuristic" or "Planning". **C.** The number of individuals in each of the categories in panel B

S4.3 Computing correlations

Correlation analysis presented in the main text focuses on decisions visited by all participants, so that each data-point used in computing correlation is based on the same population of people. In Figure S21 we show an alternative analysis, aimed to maximize the number of data-points used to compute correlation. Figure S21 shows correlations computed using decisions that were visited by at least 20% of participants.

Here, the correlation of the best-performing DU-Num model with people is $r = .89(95CI[.86, .92])$,

and the correlation of the optimal EU is $r = .72(95CI[.64, .82])$. These correlations are significantly different, with bootstrapped difference between their means of [.07.3], indicating that DU-Num predicts the aggregate population behavior better than the optimal EU model. The 95 CI of bootstrapped difference between correlations of DU-Num (a planner with the highest correlation) and Steps-Cells (a heuristic with the highest correlation) [.02.14], suggesting that the DU-Num model predicts the aggregate population better than the myopic Steps-Cells heuristic. These results remained consistent with conclusions presented in the main text as we re-ran this analysis for percentages $\in [10, 50]\%$.

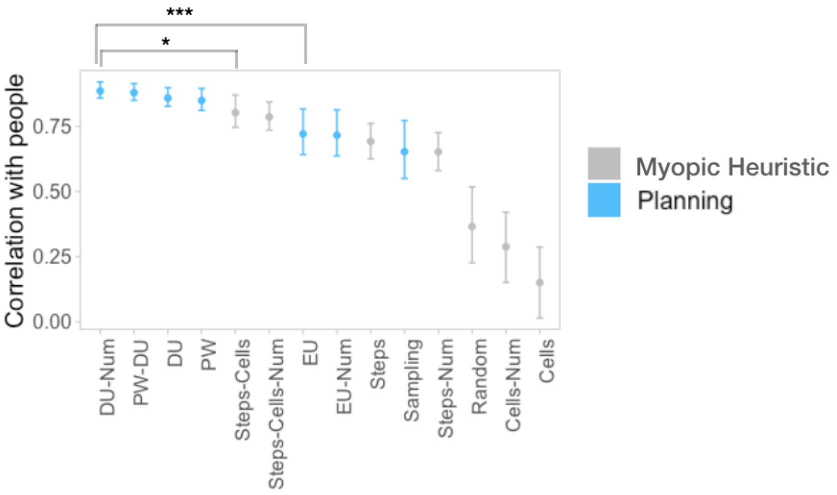

Figure S21: Experiment 1. Bootstrapped correlations of models' predictions with choice probabilities aggregated across the experimental population. The analysis includes all decisions visited by at least 20% of participants. Error bars indicate 95% confidence intervals.

Figures S24 and S25 show additional analysis in which we drop the softmax parameter, and fit the remaining parameters of each model as whichever combination of parameters agrees with the highest number of agent's choices. Then, for each model, given it best fitting parameters, we count how many times the agent's decision corresponds to the action this model suggest is an optimal choice. The results of this analysis agree with the results of our likelihood-based analysis, showing that our cognitively-inspired planners with a limited horizon explain behavior better than the EU model.

Table S1: Parameter Estimates by Model, Experiment 1

model	tau	gamma	beta	k	bits	budget	c
Cells	6.27			0.0			
Cells-Num	3.64			0.0	0.18		
DU	1.2	0.7	1.0				
DU-Num	0.56	0.51	1.0		1.9		
EU	3.0	1.0	1.0				
EU-Num	3.0	1.0	1.0		7.8		
PW	1.24	1.0	0.71				
PW_DU	1.17	0.72	1.33				
Random	1.0						
Sampling						163.58	9.69
Steps	5.02			1.0			
Steps-Cells	1.14			0.59			
Steps-Cells-Num	0.33			0.61	0.74		
Steps-Num	1.91			1.0	0.38		

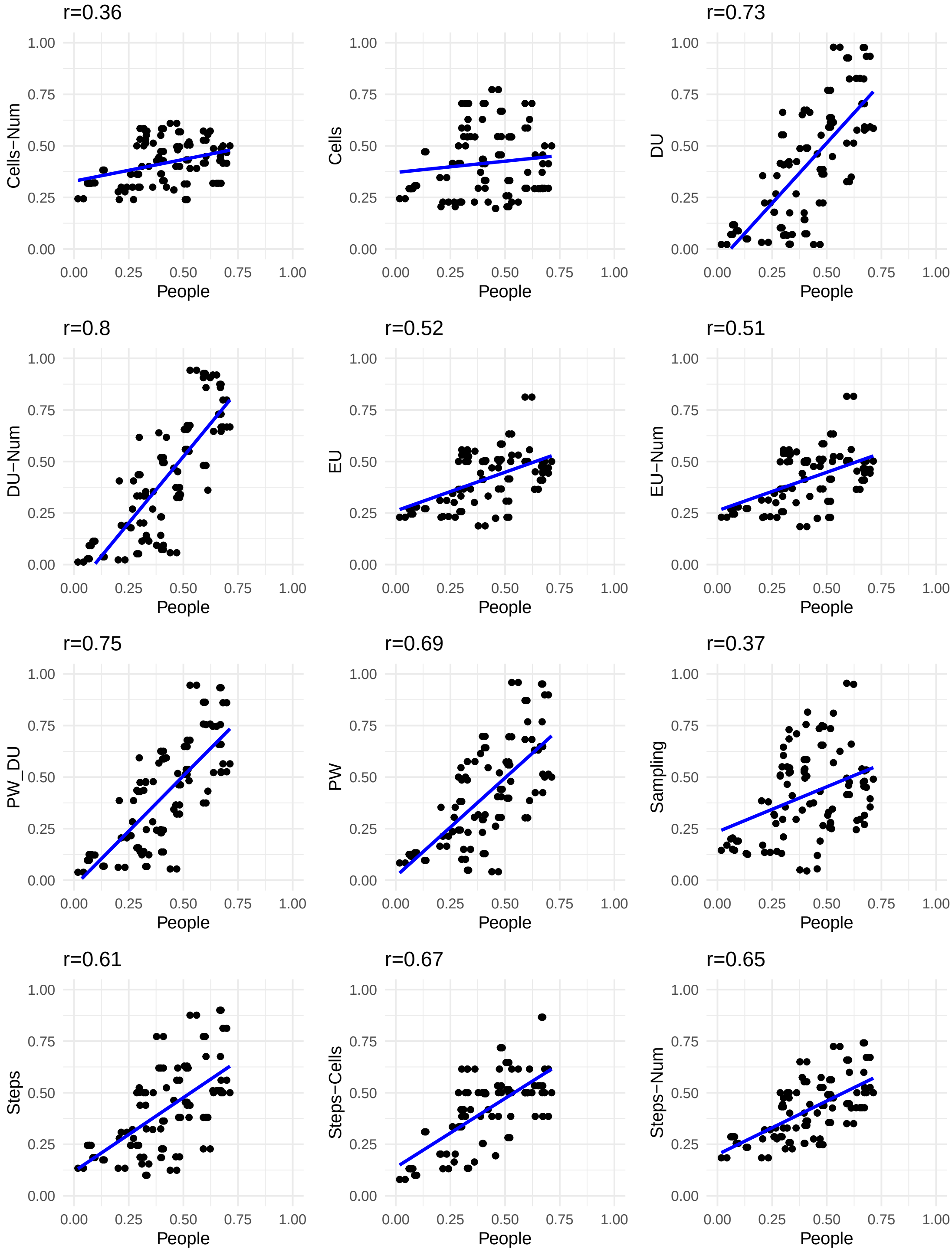

Figure S22: Experiment 1. Graded correlations between the probability of participant choice and the models' predictions, where the models are parameterized by the mean parameters fitted to the participant population, for the Initial decisions in each maze. For correlations that are significant, the correlation is shown in the title of each sub-plot.

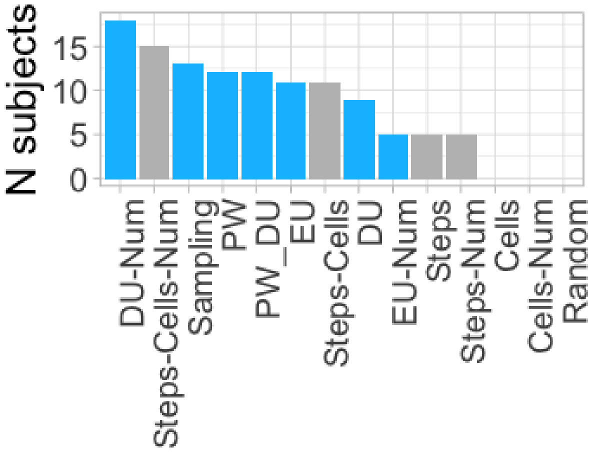

Figure S23: Experiment 1. The distribution of best-fitting models across individuals

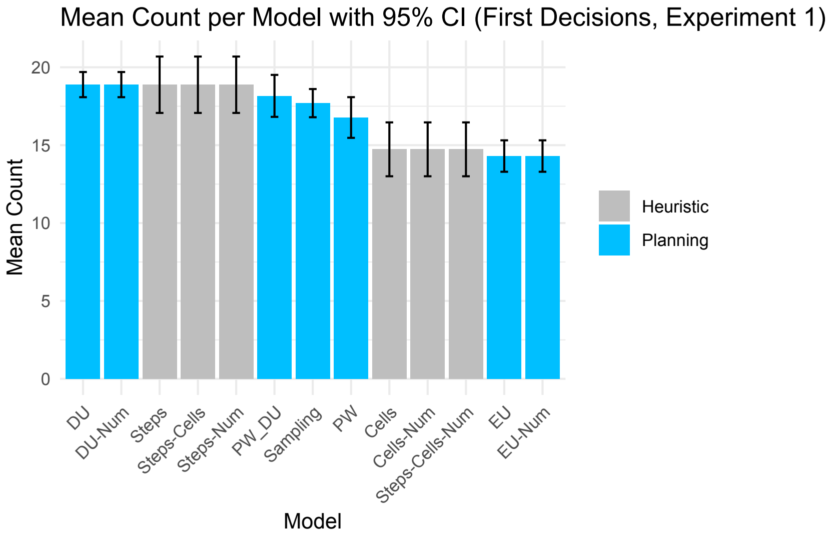

Figure S24: The number of first decisions in Experiment 1, that were optimal under each model. The counts are computed for each individual, given the best-fitted model parameters. Error bars indicate 95% confidence intervals across people.

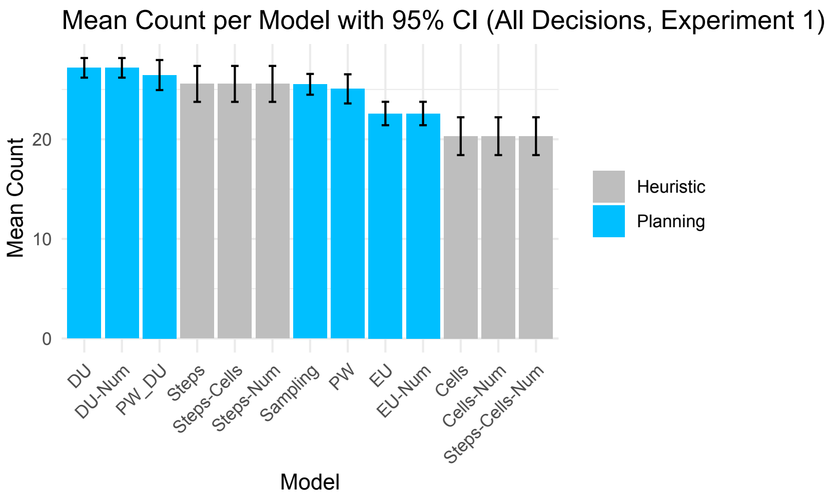

Figure S25: The number of all decisions in Experiment 1, that were optimal under each model. The counts are computed for each individual, given the best-fitted model parameters. Error bars indicate 95% confidence intervals across people.

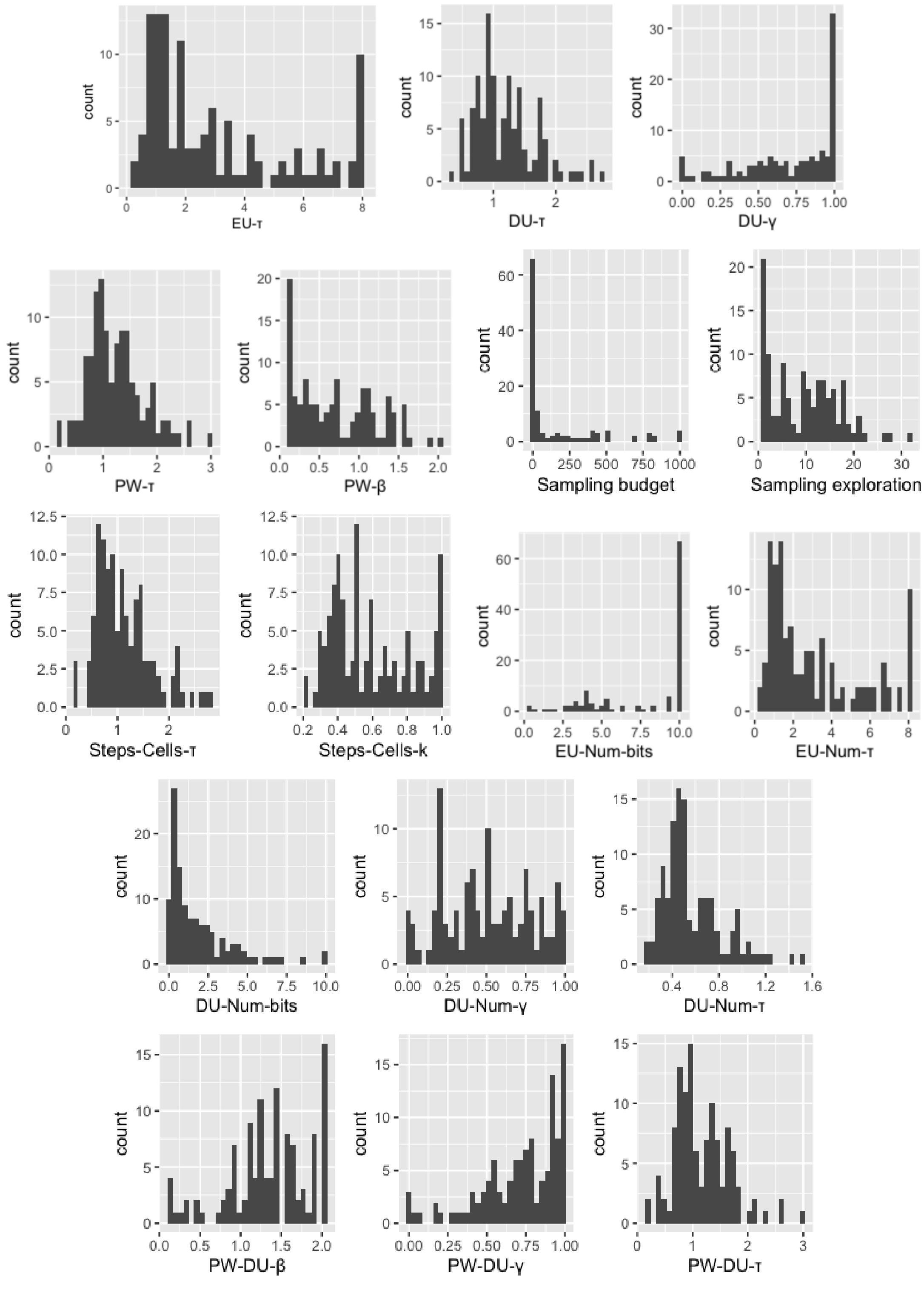

Figure S26: Experiment 1. Distribution of parameters fitted at individual level

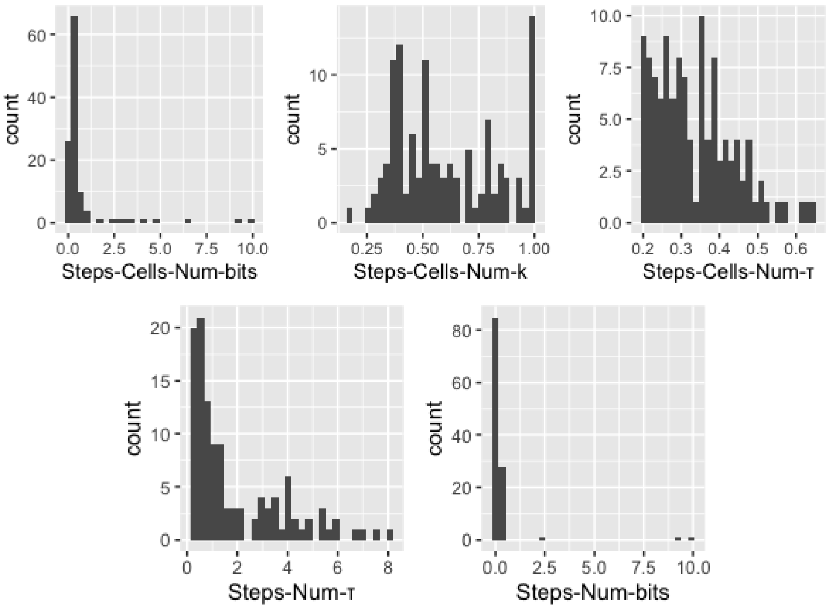

Figure S27: Experiment 1. Distribution of parameters fitted at individual level
