# Supplementary material for: Approximate planning in spatial search
Source: PLoS Comput Biol. 2024 Nov 12;20(11):e1012582. doi: 10.1371/journal.pcbi.1012582 (PMC11584085; doi:10.1371/journal.pcbi.1012582)
Supplement: S5 Appendix — (PDF) [file pcbi.1012582.s005.pdf]

S5 Experiment 2, Additional results

S5.1 Fitting models with Monte-Carlo Cross validation

We fitted models to individuals using Monte-Carlo cross-validation to illustrates variability between individuals. Figure S28 shows mean LLs of each model per move (panel A) and mean LLs per move of each individual's best fitting planner and heuristic. The labeling of individuals as "Planning" or "Not Defined" is based on non-overlapping bootstrapped 95 CI of best-fitted heuristic and planner (not the bootstrapped 95 CI for difference between means). The distribution of best fitting models across individuals is shown in Figure S29.

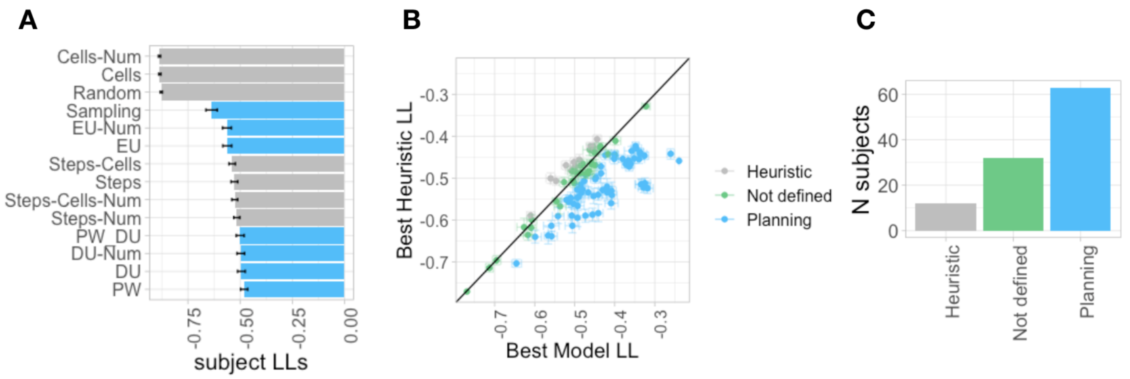

Figure S28: Experiment 2. Models fitted with Monte-Carlo cross-validation. **A.** Mean LL per decision with 95% CI, averaged over participants. **B.** Mean LL per decision for an individual's best-fitting planning model and best-fitting heuristic. Each dot represents an individual, error bars are 95%CI. Participants for whom CIs of heuristic and planning model do not overlap are labeled as "Heuristic" or "Planning". **C.** The number of individuals in each of the categories in panel B

S5.2 Computing correlations

Correlation analysis presented in the main text focuses on decisions visited by all participants, so that each data-point used in computing correlation is based on the same population of people. In Figure S30 we show an alternative analysis, aimed to maximize the number of data-points used to compute correlation, by using decisions that were visited by at least 20% of participants.

Here, the correlation of the best-performing PW-DU model with people is $r = .96(95CI[.95, .97])$, and the correlation of the optimal EU is $r = .82(95CI[.76, .86])$. These correlations are significantly different, with bootstrapped difference between their means of [.1.2], indicating that PW-DU predicts the aggregate population behavior better than the optimal EU model. The 95% CI of bootstrapped difference between correlations of PW-DU (a planner with the highest

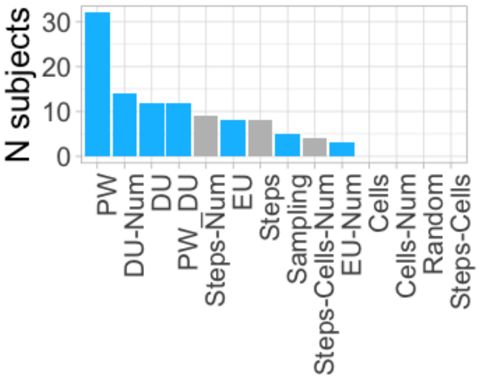

Figure S29: Experiment 2. The distribution of best-fitting planners across individuals

correlation) and Steps (a heuristic with the highest correlation) was [0.020.05], suggesting that the DU-Num model predicts the aggregate population better than the myopic Steps heuristic. These results remained consistent with conclusions presented in the main text as we re-ran this analysis for percentages $\in [10, 50]\%$.

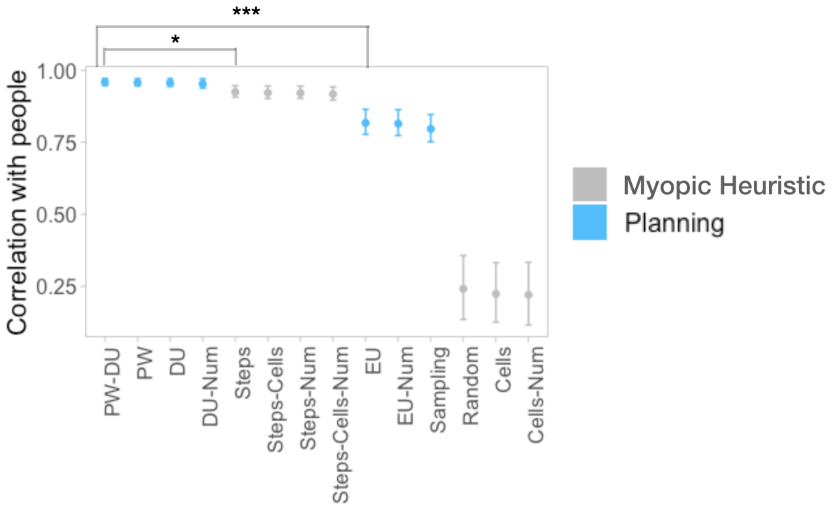

Figure S30: Experiment 2. Bootstrapped correlations of models' predictions with choice probabilities aggregated across the experimental population. The analysis includes all decisions visited by at least 20% of participants. Error bars indicate 95% confidence intervals.

Figures S32 and S33, we show additional analysis in which we drop the softmax parameter, and fit the remaining parameters of each model as whichever combination of parameters agrees with the highest number of agent's choices. Then, for each model, given it best fitting parameters,

we count how many times the agent's decision corresponds to the action this model suggest is an optimal choice. The results of this analysis agree with the results of our likelihood-based analysis, showing that planning models explain a significantly higher fraction of people's decisions.

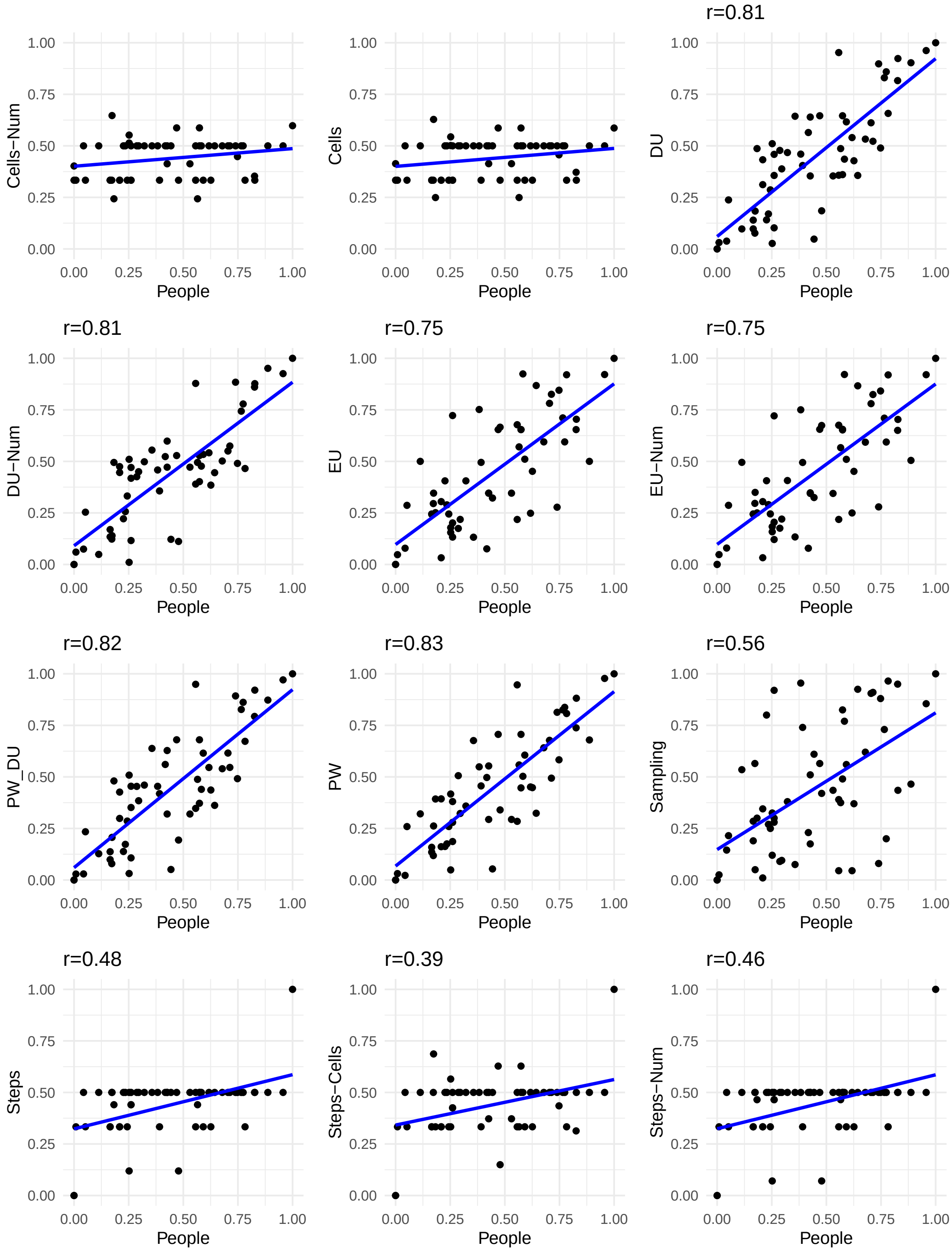

Figure S31: Experiment 2. Graded correlations between the probability of participant choice and the models' predictions, where the models are parameterized by the mean parameters fitted to the participant population, for the Initial decisions in each maze. For correlations that are significant, the correlation is shown in the title of each sub-plot.

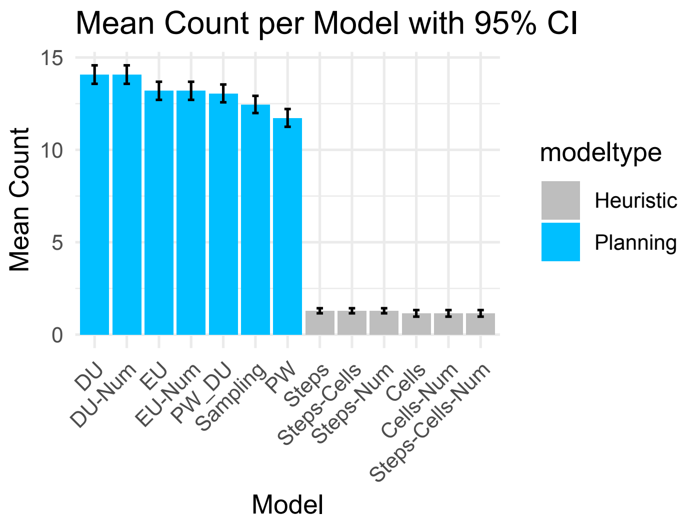

Figure S32: Experiment 2, first decisions. The counts indicate the number of an individual's decisions that were optimal under a model, given that model's best fitting parameters. Error bars indicate 95% confidence intervals across people.

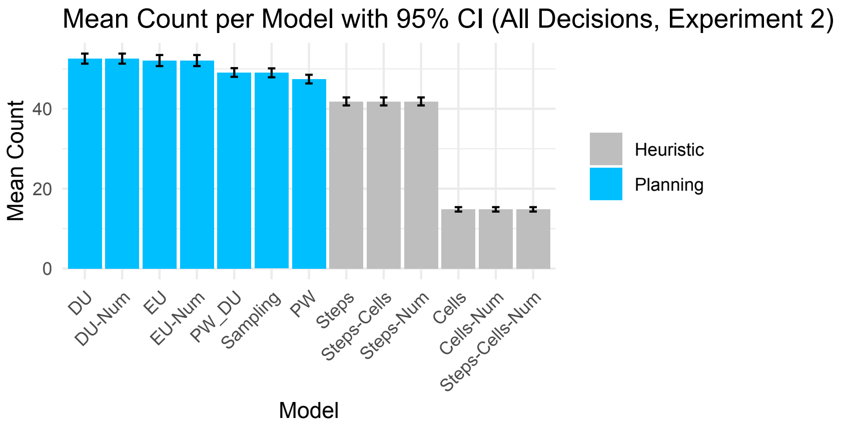

Figure S33: Experiment 2, all decisions. The counts indicate the number of an individual's decisions that were optimal under a model, given that model's best fitting parameters. Error bars indicate 95% confidence intervals across people.

S5.3 Distribution of decision times in Experiment 2

The distributions of decision-times used to compute regression models are shown in Figure S34. We removed Initial decision-times longer than 10s, and Subsequent decision-times longer then 5s for homoscedasticity needed to compute regression models.

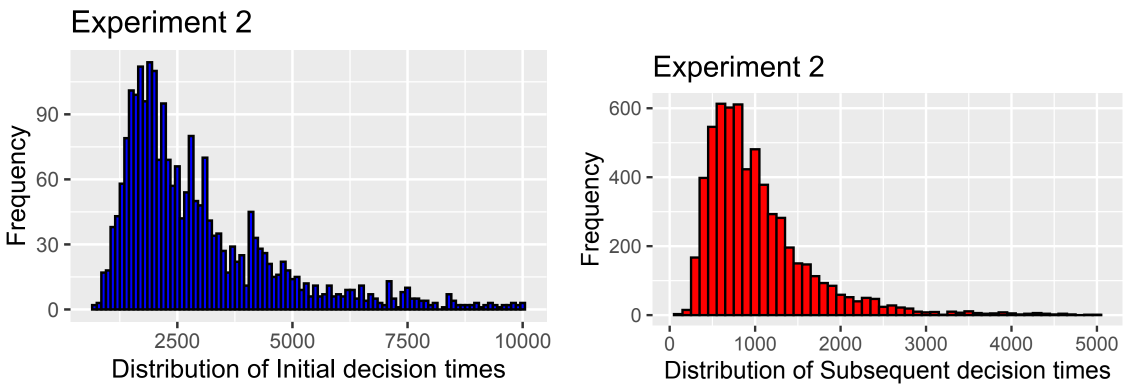

Figure S34: Distribution of initial and subsequent decision times.

S5.4 Data used to compute linear regression in Table 1

Does probability weighting explain decision times? We compute linear regression of the form $time_i = \alpha_0 + \alpha_1\beta_i + \alpha_2\tau_i + e$, where β_i and τ_i are fitted to people in model PW. In this analysis we removed data from 4 participants fitted with $\beta > 1$, so that only people fitted with $\beta in(0, 1]$ are used. Here a larger β corresponds to more accurate probability perception. In the fitted regression model we find a positive slope at β_i for Initial decision times, and a negative slope for Subsequent decision times - indicating that β in the PW model can reflect precision of computation. The results of the fitted regression model are shown in Table S2.

**Times**	p	F-statistic	α_0	Slope τ	Slope β
Initial	$< .0001$	F(2,2240)=11.54	2664	-18<br>$p = .8$	901<br>$p < .0001$
Subs.	$< .0001$	F(2,5839)=23	1080	34<br>$p = .06$	-283<br>$p < .0001$

Table S2: Linear regressions predicting decision times by parameters fitted by model PW.

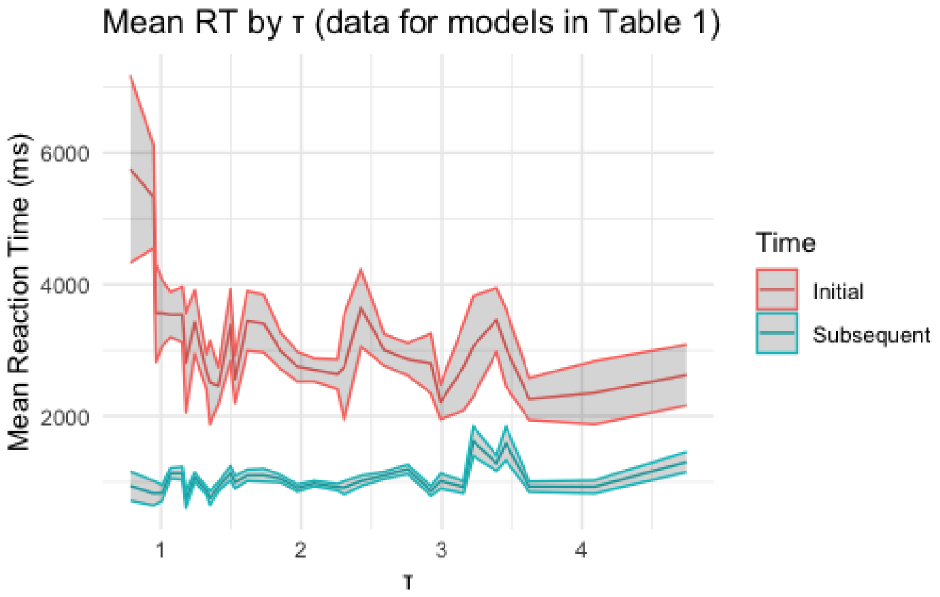

Figure S35: The response time data used to compute linear regression in Table 1, showing that people fitted with smaller τ (more optimal) have significantly longer Initial decision times, and shorter Subsequent decision times (a smaller effect).

Table S3: Parameter Estimates by Model, Experiment 2

model	tau	gamma	beta	k	bits	budget	c
Cells	5.80			0.00			
Cells-Num	1.26			0.00	0.10		
DU	0.97	0.44	1.00				
DU-Num	0.62	0.37	1.00		3.77		
EU	2.20	1.00	1.00				
EU-Num	2.18	1.00	1.00		8.64		
PW	1.14	1.00	0.35				
PW_DU	0.91	0.53	0.65				
Random	1.00						
Sampling						214.92	11.90
Steps	1.04			1.00			
Steps-Cells	0.86			0.86			
Steps-Cells-Num	0.24			0.88	0.58		
Steps-Num	0.28			1.00	0.54		

S5.5 Distribution of fitted parameters, Experiment 2

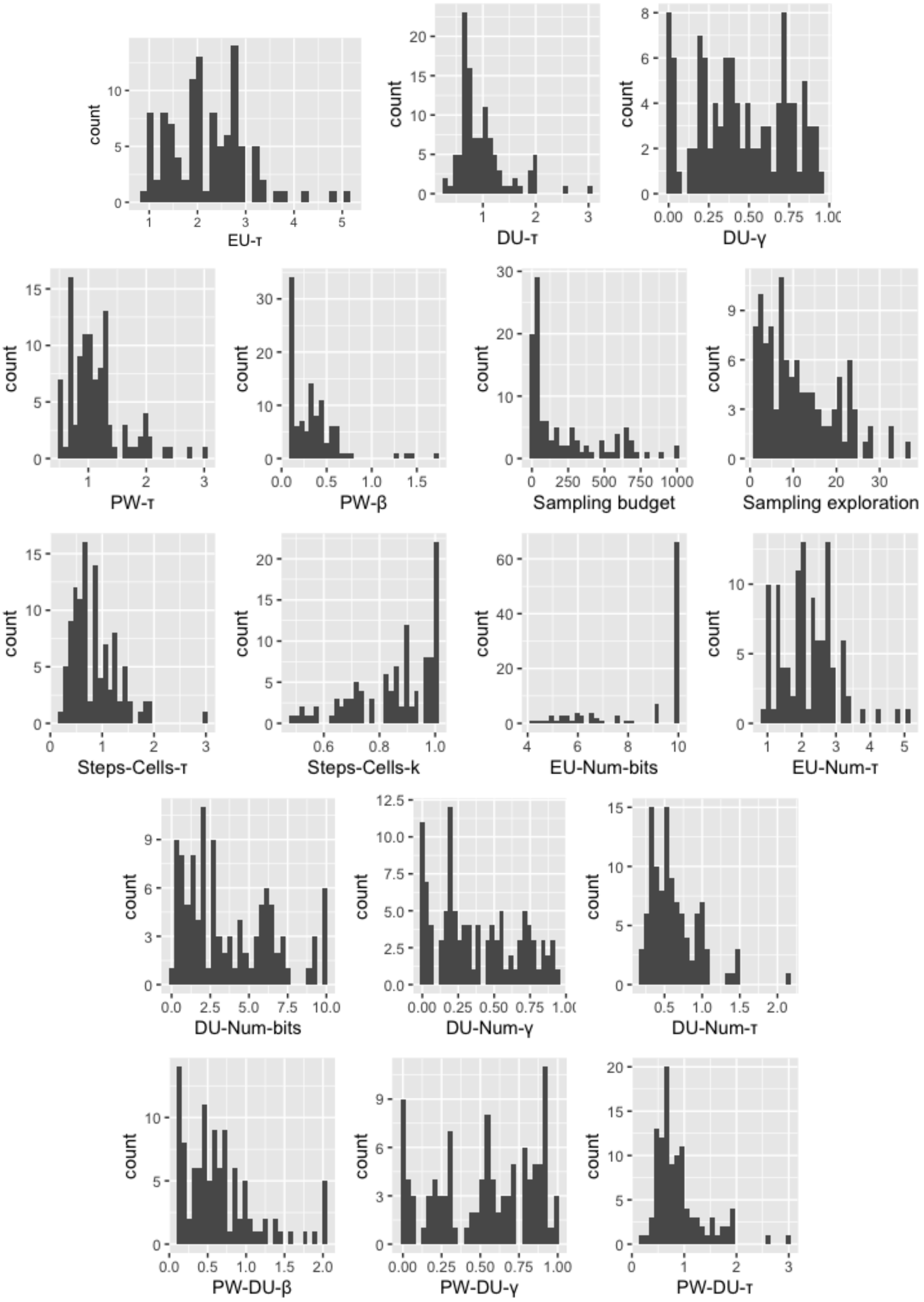

Figure S36: Experiment 2. Distribution of parameters fitted at individual level

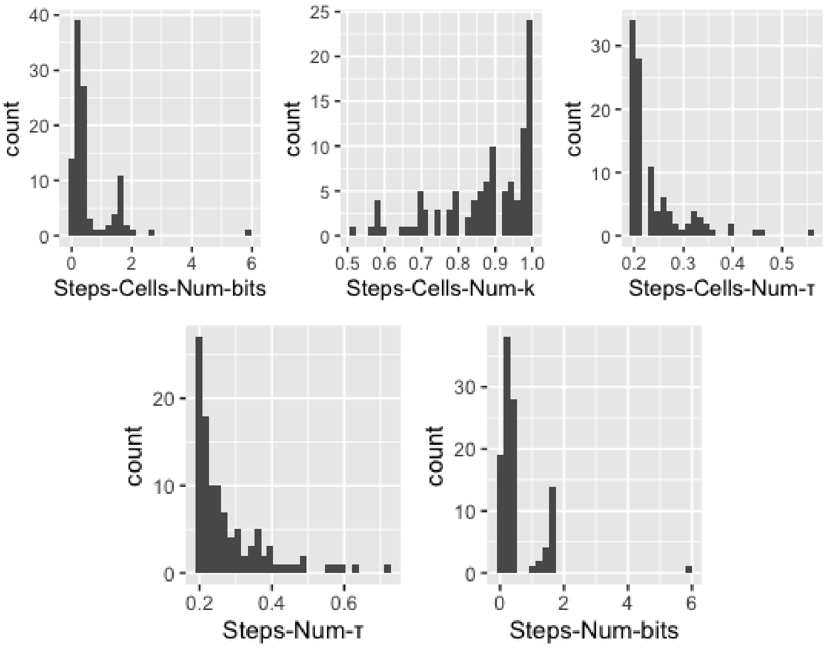

Figure S37: Experiment 2. Distribution of parameters fitted at individual level
