# Supplementary material for: Approximate planning in spatial search
Source: PLoS Comput Biol. 2024 Nov 12;20(11):e1012582. doi: 10.1371/journal.pcbi.1012582 (PMC11584085; doi:10.1371/journal.pcbi.1012582)
Supplement: S6 Appendix — (PDF) [file pcbi.1012582.s006.pdf]

S6 Maze Search Task

The MST task was extensively piloted to ensure the clarity of instructions and a sufficient amount of practice to make the task intuitive to humans. A version of MST has been used to study how do people evaluate the goodness of plans made by others [6], however human planning in MST has not yet been studied by detailed computational modeling, which is the goal of current work. An online versoin of MST can be accessed at `https://marta-kryven.github.io/experiments.html`.

Task Instructions:

Screen 1 – Instructions

Welcome to our study!
IMPORTANT
This study runs best in Firefox, on a desktop/laptop.
The study will NOT run on Safari, or a mobile device.
In this study you will look for an exit in a maze.
After this task, you will be asked to provide demographic information.
The study is expected to take about 20 minutes.
Thanks for participating!
(For brevity, we omit the informed consent statement at the end of this page)
button: [I AGREE]

Screen 2 – Instructions

INSTRUCTIONS (PLEASE READ CAREFULLY)

Your task is to exit the maze by reaching the red square in as few steps as possible.

You can move one square at a time by clicking on the white squares next to your character.

You cannot see through the walls. The squares you cannot see yet are black.

The exit is equally likely to be behind any of the black squares.

A maze looks like this:

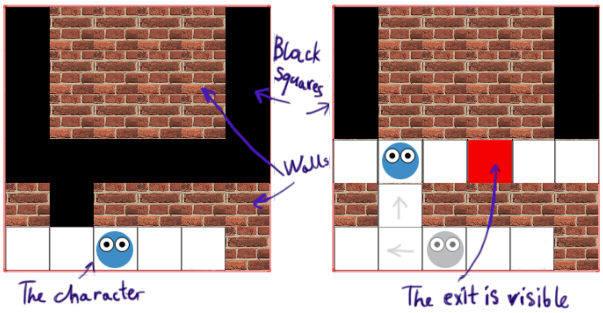

YOU CAN GET A BONUS!

Planning your path wisely will pay off.

The better you plan your path, the fewer steps you'll take and the more bonus you can earn.

A bonus of $3 will be awarded if you're in the top 20%, OR

A bonus of $2 will be awarded if you're better than average, OR

A bonus of $1 will be awarded if you're better than the bottom 30%.

button: [Let's practice!]

Screen 3 – Practice mazes

Practice Maze X of Y

Let's look at this map. There are some black squares, a brick wall, and your character.

There is ONE exit in this maze. This exit could be behind any one of the black cells.

You can move your blue character by clicking one of adjacent white cells.

Please find the exit in as few steps as possible.

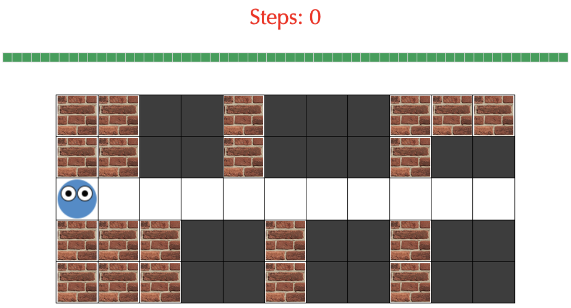

Screen 4 – Instructions Quiz

Great, you have finished Practice!

Please answer the quiz questions below to move on.

Question 1: My task is to ..

- visit every square in the maze
- see how lucky I am
- solve the mazes in as few steps as possible
- click as fast as possible

Question 2: Exits are always placed ...

- in the bottom left corner
- anywhere in one of the black cells
- in the first place I search
- in the top right corner

Question 3: Which image correctly shows parts of the maze the character has not seen yet (black squares)?

○ Image A ○ Image B

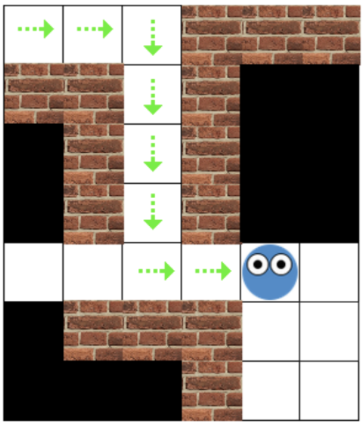

A.

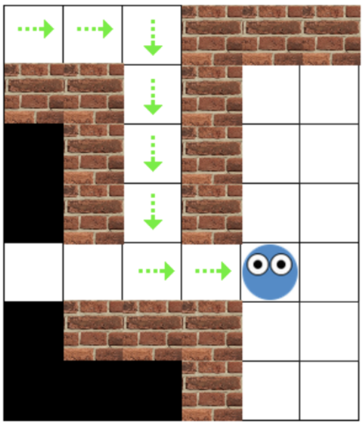

B.

button: [Submit]

Screen 5 – Experiment

Maze X of Y

Please find the exit in as few steps as possible.

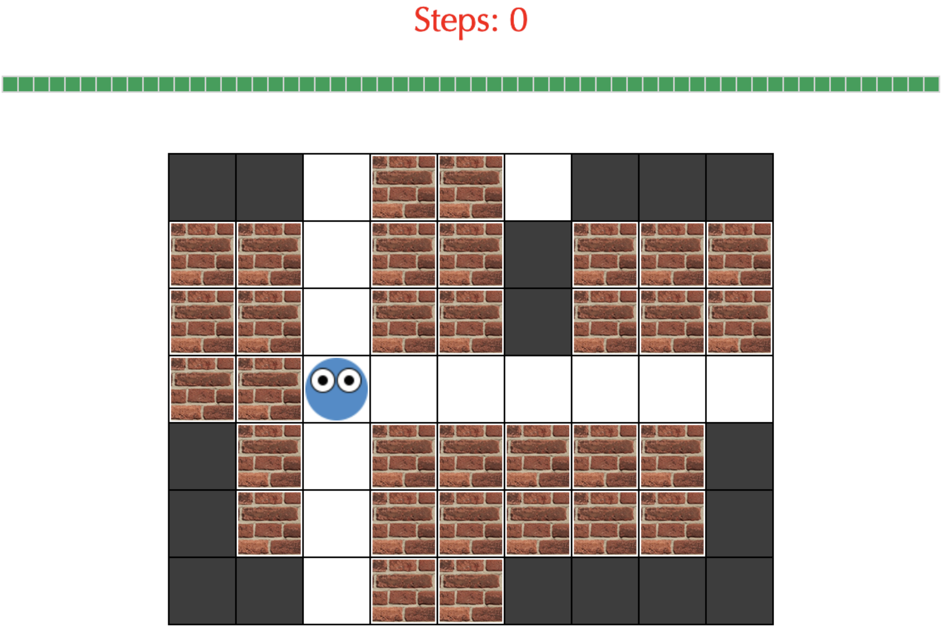

Screen 6

Thank you!

How did you make your decisions about which way to go?

Text input: [...]

button: [Submit]

Screen 7 - Demographics

Your age: [...]

Your gender: [...]

OPTIONAL: Please leave any comments about the study here, we welcome any feedback.

[...]

button: [Submit]

S6.1 Mazes Used Experiment 1

The Figures below show mazes used in Experiment 1. The mazes were presented in a randomized order. The exit location was chosen randomly at the time of experiment design. Here, the eye symbol is used to illustrate locations of the room-revealing states, this marker was not part of experiment.

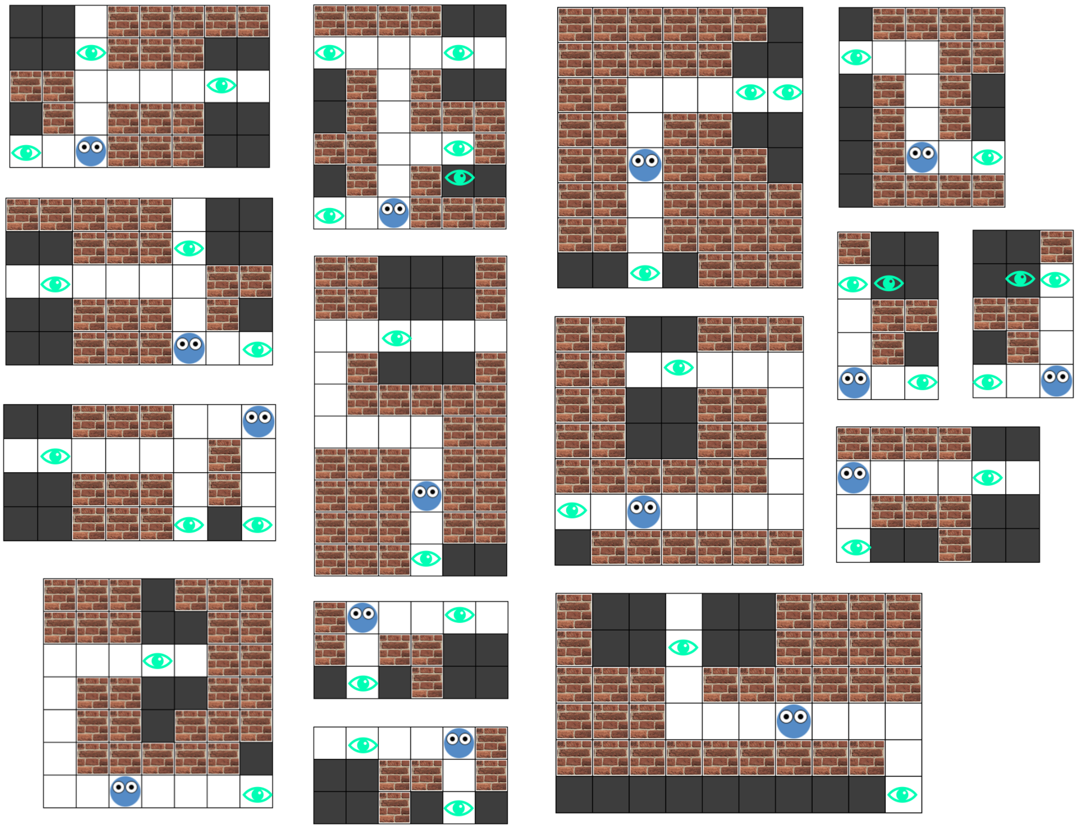

Figure S38: Mazes used in Experiment 1. The eye symbol marks the locations of room-revealing states.

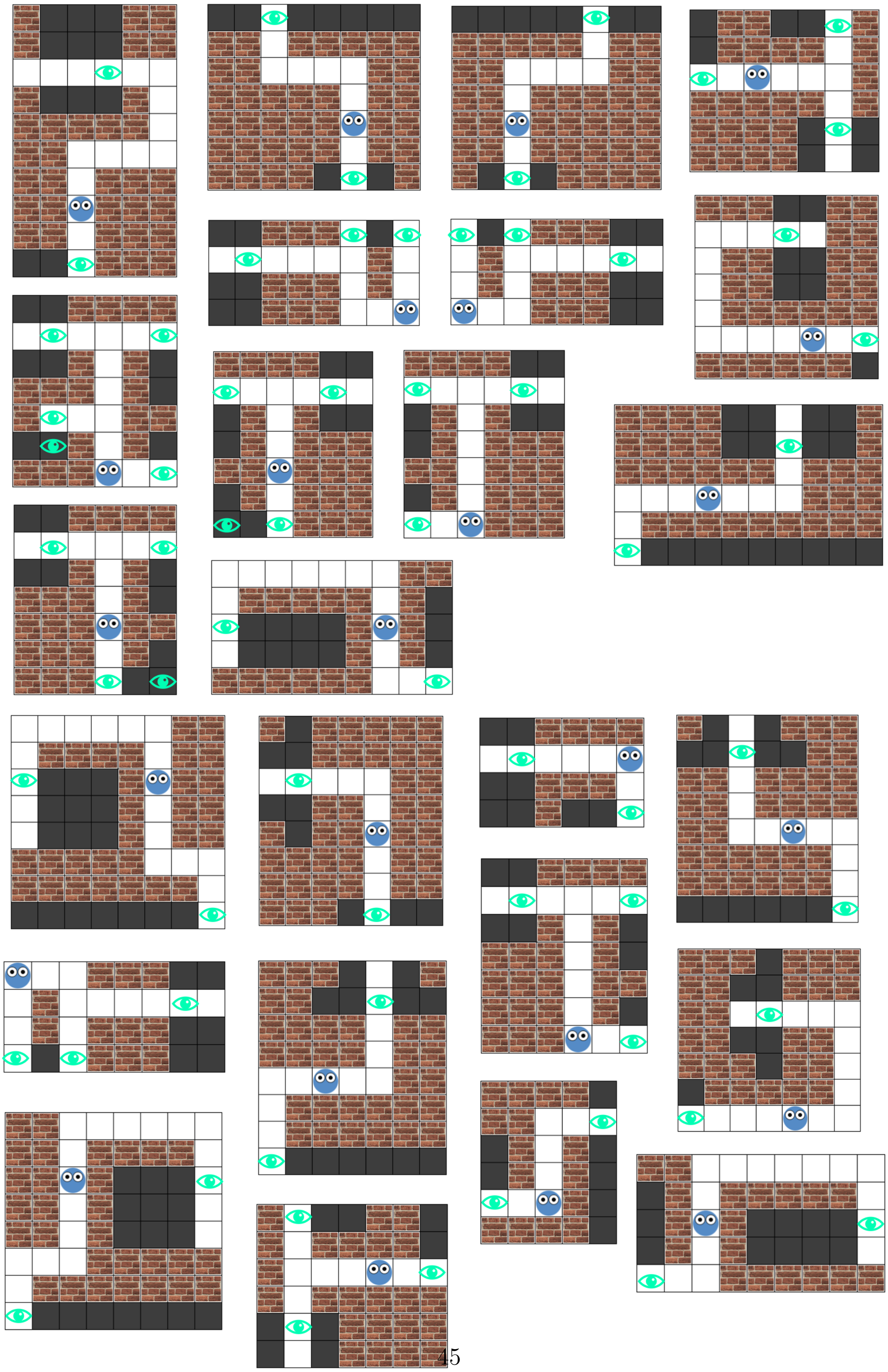

Figure S39: Mazes used in Experiment 1. The eye symbol marks the locations of room-revealing states.

S6.2 Mazes Used Experiment 2

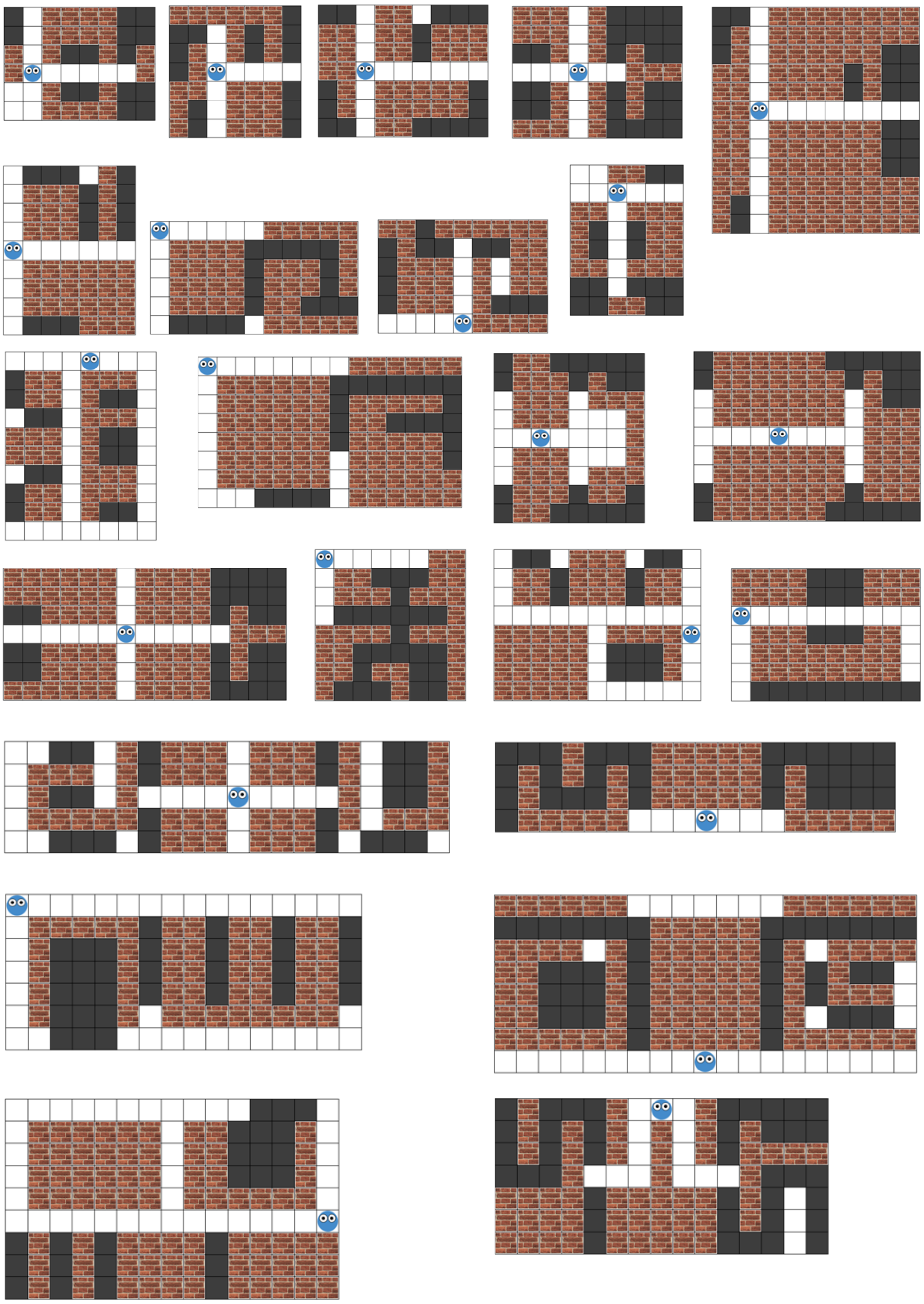

Figure S40: Mazes used in Experiment 2. The mazes were presented in a randomized order. The exit location was chosen randomly at the time of experiment design.
